# A reinforcement learning approach to airfoil shape optimization

**DOI:** 10.1038/s41598-023-36560-z

**Published:** 2023-06-16

**Authors:** Thomas P. Dussauge, Woong Je Sung, Olivia J. Pinon Fischer, Dimitri N. Mavris

**Affiliations:** 1 Aerospace Systems Design Laboratory (ASDL), School of Aerospace Engineering, Georgia Institute of Technology, Atlanta, Georgia 30332 USA; 2 Aerospace Systems Design Laboratory (ASDL), Daniel Guggenheim School of Aerospace Engineering, Georgia Institute of Technology, Atlanta, GA USA

**Keywords:** Aerospace engineering, Computer science, Fluid dynamics

## Abstract

Shape optimization is an indispensable step in any aerodynamic design. However, the inherent complexity and non-linearity associated with fluid mechanics as well as the high-dimensional design space intrinsic to such problems make airfoil shape optimization a challenging task. Current approaches relying on gradient-based or gradient-free optimizers are data-inefficient in that they do not leverage accumulated knowledge, and are computationally expensive when integrating Computational Fluid Dynamics (CFD) simulation tools. Supervised learning approaches have addressed these limitations but are constrained by user-provided data. Reinforcement learning (RL) provides a data-driven approach bearing generative capabilities. We formulate the airfoil design as a Markov decision process (MDP) and investigate a Deep Reinforcement Learning (DRL) approach to airfoil shape optimization. A custom RL environment is developed allowing the agent to successively modify the shape of an initially provided 2D airfoil and to observe the associated changes in aerodynamic metrics such as lift-to-drag (*L*/*D*), lift coefficient (*C*_*l*_) and drag coefficient (*C*_*d*_). The learning abilities of the DRL agent are demonstrated through various experiments in which the agent’s objective-maximizing *L*/*D*, maximizing *C*_*l*_ or minimizing *C*_*d*_-as well as the initial airfoil shape are varied. Results show that the DRL agent is able to generate high performing airfoils within a limited number of learning iterations. The strong resemblance between the artificially produced shapes and those found in the literature highlights the rationality of the decision-making policy learned by the agent. Overall, the presented approach demonstrates the relevance of DRL to airfoil shape optimization and brings forward a successful application of DRL to a physics-based aerodynamics problem.

## Introduction

As demand for air travel continues to grow, so are concerns regarding the environmental impacts of aviation. For aircraft, aerodynamic drag represents the main source of energy losses^1^. As such, its reduction could represent a 20 to 25% decrease in fuel burn^2^ and lead to fewer emissions. An optimization process leading to an increase in the aerodynamic efficiency of aircraft components is needed. Here, we focus on the optimization of airfoil shapes.

Relying on fluid mechanics, aerodynamic related problems exhibit non-linearity and are complex in nature^1,3^. Specifically, the problem at hand involves generating airfoils that achieve a desired performance. Formally, airfoil inverse design is described as the prediction of airfoil shapes based on given desired performance metrics^4^, making our problem an inverse design problem. Solving inverse problems is generally more difficult due to the non-injective nature of the physical phenomena^3^. In simpler terms, for a given desired performance, multiple shapes can exist^5^. Since there is a potentially infinite number of airfoil shapes, the problem is characterized by high-dimensionality. All together, airfoil shape optimization is a challenging task^6–8^. In exploring the associated high-dimensional design space, current approaches iteratively evaluate the performance of a large number of airfoils through either physical testing (wind tunnel testing) or numerical simulations (Computational Fluid Dynamics or CFD). With numerical simulations, a gradient-based or gradient-free optimizer is used to guide the search towards the optimal shape. However, these approaches are limited due to the high dimension of the design space to be explored as well as the prohibitive computational cost of running a large number of high-fidelity aerodynamic simulations^9^. Moreover, both gradient-based and gradient-free approaches are data-inefficient since they do not utilize the knowledge gained from previous experiments^3^. To cope with this, supervised machine learning approaches to computational aerodynamic problems have been developed and have proven successful in addressing these limitations^10^. In particular, a data-driven approach to the problem at hand could help alleviate the high complexity associated with the underlying physics^10^. However, since these methods rely on provided datasets, performance is dependent on their quality and found solutions are constrained by the designer’s input^3^. Hence, a data-driven approach bearing some creativity when exploring the design space is needed to successfully address the airfoil shape optimization problem.

In this work, we present a novel approach to the airfoil shape optimization problem based on Deep Reinforcement Learning (DRL). Reinforcement learning (RL) is a paradigm of machine learning focused on the discovery of optimal control^11,12^. Hence, by interacting with a provided environment, an artificial agent learns the best behavior to adopt within this environment by trying to maximize some notion of cumulative reward^12^. In this context, by modeling the airfoil shape design as a Markov decision process (MDP), RL can address the limitations of past approaches. Although RL has proven extremely successful in addressing game-based problems, we demonstrate here that this approach is also suited for an aerodynamics problem traditionally addressed from a physics-based perspective. Given certain flow conditions, the goal here is to train an artificial agent to generate an optimal airfoil that maximizes a specific aerodynamic objective by modifying the shape of an initially provided airfoil.

Previous work has focused on a RL approach to the airfoil shape optimization problem. However, past studies were limited by discrete action spaces^11^, limited design space exploration^7^ or the need for transfer learning, enhancing the learning process^8^. Here, we show that provided a custom environment, an RL-based agent is able to effectively explore a high-dimensional design space for multiple aerodynamic objectives. We also investigate the impact of shape parametrization on the learning process and benchmark our DRL approach to a classical simplex method. Finally, the artificially produced shapes are compared to existing airfoils: the strong geometric resemblance found emphasizes the rationality of the policy learned by the agent.

The background associated with the airfoil shape optimization problem as well as the limitations of past and current approaches is presented in “Background” section. Section in “Problem definition” provides a clear definition of the problem at hand and identifies the gaps the presented research aims to address. Finally, the investigated DRL approach is detailed in Section IV and results are brought forward in section “Results”.

## Background

Airfoil shape optimization has been an active research topic since the 1960s^7^. Numerous techniques have emerged to address this problem and are currently widely used. These methods can be broadly classified into gradient-based and gradient-free optimization methods. Recently, the use of new techniques based on supervised machine learning have proven successful in solving non-linear and high-dimensional problems, making them suited for aerodynamic problems^1,13–15^. Moreover, such data-driven approaches stem from the need for more data-efficient methods, which leverage knowledge from past experiments^3^. However, supervised learning methods are limited because they rely on user-provided datasets^3^. A reinforcement learning approach can address these limitations and provide a data-driven approach to a high-dimension exploratory problem. Such approach to the airfoil shape optimization problem remains limited, as suggested by the literature^6–8,11^. However, it has been demonstrated that when coupled with deep neural networks, RL exhibits unprecedented efficiency in learning the optimal control of complex dynamic systems^1^, which makes this approach a promising candidate to address the airfoil shape optimization problem.

### Current airfoil shape optimization techniques

**Figure 1:**
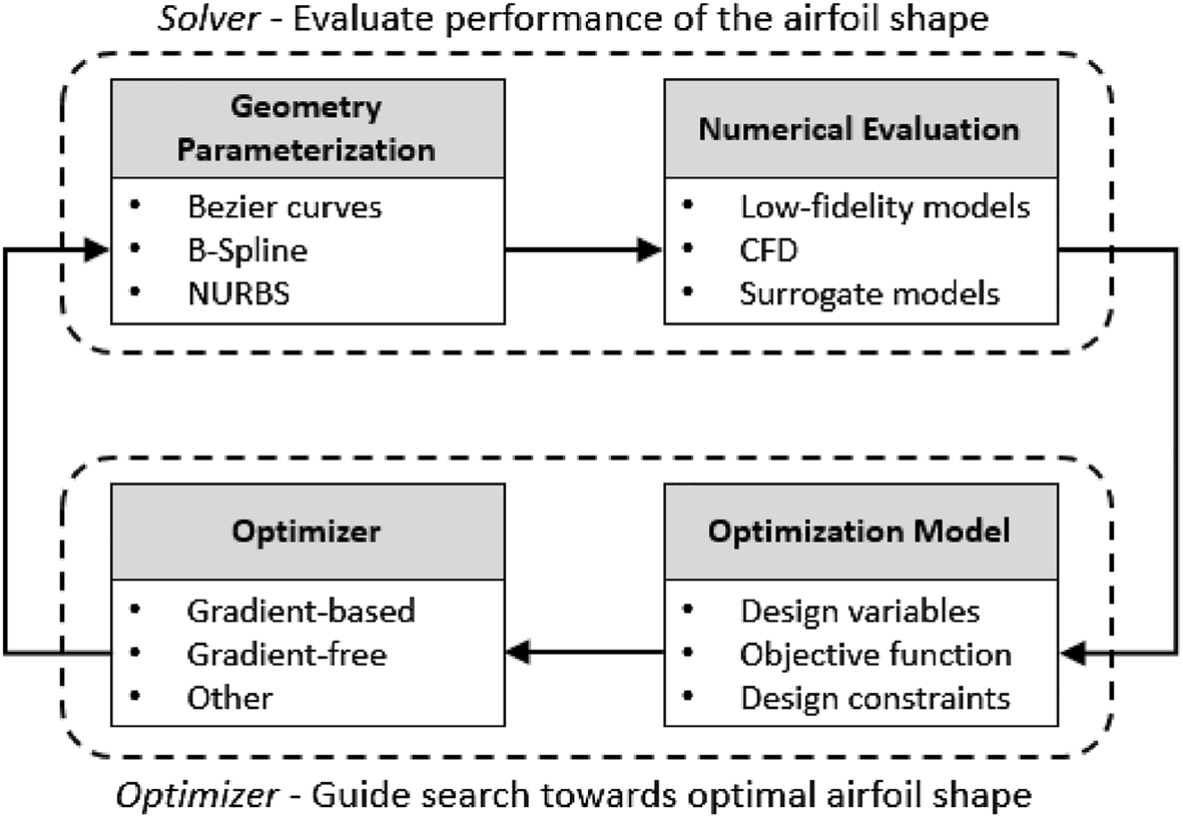
Current airfoil shape optimization workflow (modified from^8^).

Current aerodynamic shape optimization schemes rely on (i) a solver or response surface equations (RSE) that capture the physics of the tested shape, and (ii) an optimizer to guide the search towards the optimal shape within the design space. This process is represented in Fig. 1. The aerodynamic solver can either be a physics-based tool, having a low or high degree of fidelity, or surrogate models of these tools, which can help decrease computational costs and run times. Coupled with the aerodynamic solver is an optimizer which encapsulates the optimization problem.

Two main optimization approaches have emerged to tackle the airfoil shape problem. Gradient-based methods rely on calculating the gradient $\nabla _{x}J$ of a cost function *J* with respect to its design variables *x* to guide the search towards a global optimum. For airfoil shape optimization, the cost function *J* can be a metric of interest associated to the airfoil, such as the lift-to-drag ratio *L*/*D*, and the design variables are control points that describe the airfoil 2D shape. Significant research has been conducted on gradient-based methods applied to aerodynamic problems. A detailed comparison of these methods can be found in^16,17^. Within gradient-based methods,^18^ introduced the adjoint method in fluid dynamics, demonstrating that computational cost is independent of the number of design variables. Further research has led to the discrete adjoint method^19,20^, which is widely used in fluid dynamic optimization. Hence, advantages of gradient-based optimization in addressing airfoil shape optimization are low computational costs in large design spaces^2,7,8^ and a track record of successful implementations in aerodynamics^2,6^. However, some drawbacks exist to this approach, such as a tendency to converge on local optima and a high sensitivity to the starting point^6–9,21^, bad efficiency for non-linear cost functions^7^ and the need for continuous shapes (the gradient of the shape must exist in all points)^2^.

Gradient-free methods address some of these issues: they are better at finding the global optimum^2^ and are well suited for complex optimization tasks (non-linear and non-convex functions)^2,7^. Within gradient-free methods, Genetic Algorithms (GA) modified for aerodynamic optimization have been developed to tackle the limitations of gradient-based methods^22,23^. The benefits of gradient-free methods come at the cost of more complex methods having higher computational costs (compared to gradient-based approaches), poor constraint handling abilities and limitations on the number of design variables handled^2^, resulting in low convergence speeds when coupling gradient-free methods with high-fidelity solvers^6,8,9^. For instance,^17^ demonstrates the computational expense of using the NSGA-II genetic algorithm for RANS-based optimization. To mitigate this expense, investigations of parallel deployment of GAs for airfoil optimization have been conducted, as presented in^24^. Despite its computational cost, there have been applications of GAs to airfoil shape optimization, such as optimization of laminar flow regions over an airfoil using Multi-Island GA (MIGA) in^25^. Finally, another class of gradient-free methods, namely Particle Swarm Optimization (PSO), has been applied to airfoil shape optimization, as presented in^26–28^. Table 1 summarizes the advantages and disadvantages of both gradient-based and gradient-free methods.

**Table 1:** Gradient-based versus gradient-free methods.

	Gradient-based	Gradient-free
Advantages	Low-computational cost	Good at finding global optimum
	Widely used method in aerodynamics	Well suited for complex functions (non-linear)
Drawbacks	Poor efficiency for non-linear function	
	Convergence on local optima	High computational cost
	Sensitive to starting point	Limitation on number of design variables
	Requires continuous function	Low convergence speed when coupled with CFD
	Inability to use past optimum data	Inability to use past optimum data
Popular	Finite difference method	Multi-objective Genetic Algorithms (MOGA)
Algorithms	Adjoint method	Multi-objective Particle Swarm Optimization (MOPSO)

Additionally, recent studies have also tackled the impact of airfoil parametrization on optimization efficiency, demonstrating that a deep-learning approach to airfoil parametrization improves optimization efficiency^9^.

Hence, there is a compromise between using gradient-based and gradient-free approaches which lies on the trade-off between computational cost and (i) the ability to handle complex non-linear functions and (ii) the effectiveness at finding a global optimum. Coupling any of these techniques with a high-fidelity solver results in prohibitively low convergence speeds. Moreover, for both gradient-based and gradient-free approaches a new optimization problem is posed with each new search. To increase computational efficiency, data-driven approaches which utilize the knowledge accrued from past experiments must be investigated^3^. In this context, machine learning based methods may address these limitations.

### Machine learning for shape optimization

Learning is critical in any design process^29^ and areas where artificial intelligence can be useful in design are plenty^30^. Here, we are interested in the design of optimal airfoils. Innovative techniques based on machine learning (ML) have proven successful at addressing aerodynamic optimization and allowed for increased predictive and control capabilities^10^. Though the knowledge associated with these techniques is not recent, their application is, owing to increased computational capabilities and available data. Indeed, most machine learning techniques rely on large sets of data and sufficient computational power to allow an intelligent agent to learn. Deep neural networks (DNN) in particular, are well suited for non-linear problems of high-dimensionality since they are universal approximators and can in theory solve any problem represented by a function^1^. As an example, DNNs have been applied to predict turbulent flows, proving to be more accurate than RANS (Reynolds-averaged Navier-Stokes) models in some cases^13^. The literature is rich with illustrations where machine learning methods, when coupled with current airfoil shape optimization methods, have surpassed other gradient-based and gradient-free approaches in terms of complexity of the tasks learned and learning speeds^1^. It is worth noting that most successful implementations of machine learning approaches for airfoil design reside in infusing ML to specific portions of the optimization workflow, and should not be viewed as a complete replacement to current state-of-art airfoil optimization^31^, but rather as an alternative approach to the optimizer (Fig. 1).

There have been successful implementations of supervised neural network (using labeled data to predict the label of unlabelled data) together with gradient-based and gradient-free techniques to the airfoil shape optimization problem. In^14^, a generative adversarial network (GAN) is used to reduce the dimensionality of the airfoil shape optimization problem resulting in a reduced number of evaluations required to find the optimal solution compared to other algorithms. In^15^, a conditional generative adversarial network (CGAN, semi-supervised learning) was used to train an artificial agent to generate realistic airfoil shapes having specified aerodynamic characteristics such as lift-to-drag ratio. These findings demonstrate the relevance of machine learning at solving complex aerodynamic problems and more specifically their relevance to the airfoil shape optimization problem.

However, the machine learning techniques illustrated above rely on extensive datasets from which they learn. From this, two issues can be mentioned: (i) such substantial data is not always available, depending on the problem at hand, and (ii) found solutions are constrained by the provided dataset i.e., the learned behavior will reflect from the data, meaning that no radically different behavior (unthought of by human intuition) can be produced by this type of learning^3^. Another paradigm of machine learning, namely reinforcement learning, which does not rely on user-provided data, can alleviate these issues.

### Markov decision processes and reinforcement learning

**Figure 2:**
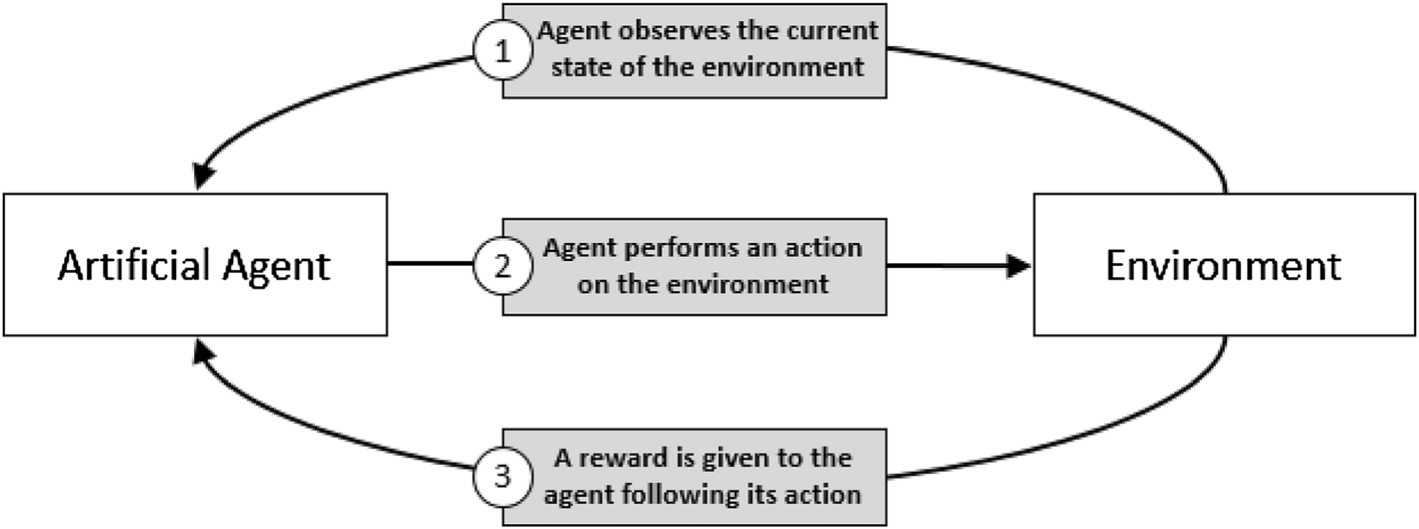
Reinforcement Learning Framework (adapted from^7,12^).

The field of machine learning is currently comprised of three paradigms: supervised, unsupervised and reinforcement learning. In a supervised setting, learning is performed on a training set of labeled examples provided by a knowledgeable external supervisor with the goal of predicting the label of unlabeled inputs. Unsupervised learning is used to find the structure hidden within a collection of unlabeled data^12^. In reinforcement learning (RL), an artificial agent is tasked with learning the optimal behavior to follow within an environment by trying to maximize some notion of cumulative reward which is given after taking an action in this environment^1,6,7,12^. In comparison to supervised and unsupervised learning, reinforcement learning does not require large sets of user-provided data for its learning process. Rather, the agent gathers experience by interacting with the provided environment, and exploits the obtained knowledge to improve its behavior. The agent interacts with the environment through three signals of information: (i) an observation of the current state of the environment, (ii) an action performed by the agent on the environment, justified by the observation, and (iii) a reward given to the agent after taking said action (shown in Fig. 2).

Reinforcement learning became popular through its application to widely played games. For example, *AlphaGo* is an algorithm that was trained through RL and achieved superhuman proficiency at playing the game of *Go*^32^. In this example, the environment is the board grid and the observation is the current placement of the game pieces on the board at a given time. The action of placing a game piece at a given position on the board is followed by an increase or decrease in the probability of winning the game, which represents the immediate reward of the previously taken action. The classic *Atari 2600* video games have also been used to experiment with RL^33^. The agent obverses the state of the game in the same way a person would observe the video game screen and selects an action to perform on the joysticks and buttons available (up/down, right/left, shoot/shield). Following this action, a reward based on the increase or decrease in the game score is given to the agent. RL has been applied to these particular games because they provide adequate frameworks for its successful implementation. More generally, RL is agnostic to the details of the environment and is applicable to any time-dependent process satisfying a state/action/reward interface. Such an interface is defined as a *Markov decision process* (MDP).

A finite MDP is defined by a 4-tuple (*S*, *A*, *P*, *R*) in which *S* represents the finite set of all possible states the environment can be in, *A* is the finite set of all actions the agent can take, *P* is the state transition probability matrix and *R* is the set of all possible rewards. At a given time-step *i*, the agent observes the environment that is in state $s_{i} \in S$, chooses an action $a_{i} \in A$ to perform on the environment, which transitions to state $s_{i+1}$ with a probability $p(s_{i+1},|s_{i},a_{i})$ awarding the agent a reward $R_{i+1} = r(s_{i}, a_{i}, s_{i+1})$^12,34^. The choice of the agent to take action $a_{i}$ when the environment is in state $s_{i}$ is dictated by a *policy* $\pi _{i}: S \rightarrow A$. The goal is to find the optimal policy which will maximize the agent’s cumulative reward over the long run. In this context, RL methods are used to approximate an optimal policy, details of which are provided here after.

The sequence of state-action defines the trajectory $\tau$ followed by the agent:

1 $$\begin{aligned} \tau = (s_{0}, a_{0}, s_{1}, a_{1}, ...) \end{aligned}$$

The agent’s goal is to maximize the cumulative reward it receives over the trajectory. The expected cumulative reward at time-step *t* is defined as^12^:

2 $$\begin{aligned} G_{t} = \sum _{k = 0}^{T}\gamma ^{k}R_{t+k+1} \end{aligned}$$

where *T* is the total length (in terms of iterations or time steps) of the game, $R_{i}$ is the immediate reward received at time step *i* and $\gamma \in [0, 1]$ is a discount factor taking into account the impact of future rewards on the current expected reward. In order to maximize the cumulative reward, the agent must learn the optimal behavior when in a given state, defined as the policy $\pi$, which maps a state to probabilities of selecting each possible action when in that state. The way in which the agent approaches this optimal behavior can be classified into two methods^7,12^.

1. In *value-based* methods, the agent learns how to best estimate a value function, which gives the expected reward when starting in state *s* and following the policy $\pi$ after. By selecting the action having the highest value, the agent follows the optimal policy. Two value functions can be defined, namely:
  (i) The *state-value function* for policy $\pi$, which gives the expected return when starting in state *s* and following the policy $\pi$ after
    3 $$\begin{aligned} v_{\pi }(s) = {\mathbb {E}}_{\pi }(G_{t}|s_{t} = s) \end{aligned}$$
  (ii) The *action-value* function for policy $\pi$, which gives the expected return when starting from *s*, taking action *a* and thereafter following $\pi$
    4 $$\begin{aligned} q_{\pi }(s, a) = {\mathbb {E}}_{\pi }(G_{t}|s_{t} = s, a_{t} = a) \end{aligned}$$
    The Bellman equation provides a relationship between the value of a given state and the values of its successor states. For the *state-value* function, this equation is written as:
    5 $$\begin{aligned} v_{\pi }(s) = \sum _{a}\pi (a|s)\sum _{s',r}p(s',r|s,a)[r + \gamma v_{\pi }(s')] \end{aligned}$$
    The optimal *state-value* function, denoted as $v_{*}$ is defined as
    6 $$\begin{aligned} v_{*} = \max \limits _\pi v_{\pi }(s) \end{aligned}$$
    and has an associated Bellman equation, the Bellman optimality equation
    7 $$\begin{aligned} v_{*} = \max \limits _a\sum _{s',r}p(s',r|s,a)[r + \gamma v_{*}(s')] \end{aligned}$$
    Solving this last equation yields the optimal *state-value* function, which in turn yields an optimal policy^12^ (there can be more than one optimal policy). It is worthwhile to note that one of the main approaches used in finding the optimal policy through *value-based* methods is Q-learning, which relies on estimating the optimal action-value function *q*, yielding breakthrough results when combined with deep neural networks in^33^.
2. In *policy-based* methods, the optimal policy is sought by directly optimizing the policy $\pi$ without estimating an optimal value function. This class of methods offers advantages over the *value-based* approach: (i) better capabilities in handling high-dimensional action spaces, (ii) suited for both continuous and discrete action spaces (where *value-based* methods perform poorly on continuous action spaces), and (iii) smoother convergence properties (convergence on local minima is however possible)^6,7^. Similarly to *value-based* methods, a function based on the expected cumulative reward over the whole trajectory is needed to evaluate the performance of a given policy:
  8 $$\begin{aligned} J(\theta ) = {\mathbb {E}}_{\tau \sim \pi _{\theta }}[G(\tau )] \end{aligned}$$
  Optimal parameters $\theta ^{*}$ of the policy which maximize $J(\theta )$ are sought, such that:
  9 $$\begin{aligned} \theta ^{*} = arg \max \limits _{\theta }{\mathbb {E}}_{\tau \sim \pi _{\theta }}[G(\tau )] \end{aligned}$$
  Calculation of the gradient of $J(\theta )$ and further details on how the optimal policy parameters are found is not given here, but can be found in^6^.

In summary, airfoil shape optimization is an iterative process and as with any design process, learning can be performed from a series of observations/experiences^29^. Moreover, due to the complexity of the underlying physics of the problem at hand, a data-driven approach should be pursued allowing to explore high-dimensional design spaces, while not relying entirely on user-provided data and having some exploratory behavior. Here, we approach airfoil design as a series of time-dependent states/actions/rewards, best described as a Markov decision process. Under these conditions, reinforcement learning is a relevant and promising approach to solving the MDP associated with the airfoil shape optimization problem. It is important to emphasize that the novelty of the approach presented hereafter resides in formulating the airfoil design process as a Markov decision process, leading to the relevant use of Deep Reinforcement Learning to successfully train an artificial agent capable of guiding the design of optimal airfoil shapes.

### Reinforcement learning for shape optimization

The theory behind reinforcement learning (RL) is not new, as interest in this particular paradigm of machine learning started around 1979^12^. The application of RL has traditionally been limited to low-dimensional problems. However, when combined with deep neural networks (DNN) and exploiting the feature extraction capabilities of the latter, *deep* reinforcement learning (DRL) exhibits remarkable efficiency at tackling high-dimensional problems^6^. This stems from the fact that artificial neural networks are universal function approximators and can be used in the context of RL to efficiently approximate an optimal policy. As fluid mechanics exhibit high-dimensionality and non-linear behaviors, DRL appears as a suitable candidate for solving complex problems within this particular field^1^. Nonetheless, applications of DRL to fluid mechanics remain limited, as noted by^6^ and^7^. To the best of our knowledge, only three applications of RL to the airfoil shape optimization problem have been published.

In^11^, RL is applied to the problem of morphing airfoils, i.e., in-flight shape modification of the airfoil to best meet performance targets at a given flight regime. Morphing of the airfoil is permitted through three degrees of freedom, namely chord, thickness and camber of the airfoil, leading to a shape that corresponds to specified goal requirements such as achieving values for the lift coefficient *C*_*l*_, drag coefficient *C*_*d*_ and moment coefficient *C*_*m*_. Q-learning is used to learn the optimal change in shape policy and it is shown that an optimal policy can be learned after 3000 episodes having a 100% success rate (the produced shape meets all performance targets). However, the presented approach does have limitations. The agent’s degrees of freedom are the airfoil’s chord and overall camber and thickness, and not local camber and thickness at a selected chordwise position. Additionally, the changes permissible are discrete (i.e., the action space is discrete), which justifies the use of Q-learning since it can only handle discrete action spaces. As a result, this approach is also limited by the limitations of value-based methods, which perform poorly on high dimensional design spaces. Moreover, boundaries are set for the acceptable change in airfoil thickness and camber: the agent is negatively rewarded if exploring beyond those limits. Overall, in this approach the exploratory freedom available to the agent is relatively low. We believe an approach bearing localized and continuous changes to the airfoil shape would allow to fully explore the high dimensional design space associated with the problem at hand and leverage the full generative capabilities of RL.

A DRL approach to aerodynamic shape optimization of missile control surfaces is presented in^8^. The goal is to find the missile configuration having the highest possible lift-to-drag ratio, under both aerodynamic and geometric constraints, with the geometry having to be tested through CFD. The research presents a novel approach to this optimization problem, based on reinforcement learning and transfer learning. A first neural network is trained through RL using a low-fidelity aerodynamic solver. This allows for the neural network to quickly learn an optimal policy. The network parameters are then transferred over to another neural network which is trained using a higher fidelity solver. The novelty of this approach lies in the use of transfer learning, where the knowledge from one trained DNN (trained on the low-fidelity solver) is transferred over to another untrained DNN (the DNN based on the high-fidelity solver) through sharing of network parameters. This leads to reduced computational times: a decrease of 62.5% in CFD calls (high-fidelity solver) is observed, compared to a non-transfer learning approach. Although this research is not focused on airfoil shapes, the described RL approach for an aerodynamic optimization problem is worth acknowledging. More specifically, this research is not concerned with intrinsic airfoil parameters such as thickness and camber, but rather with higher level geometry parameters such as chord length, sweep and span of the missile’s wing tip. The impact of the positioning of these wing tips on the missile’s overall aerodynamic performance is investigated. Finally, these design variables have fixed bounds, thus limiting the exploratory capabilities of the proposed approach.

In^7^, a neural network is trained through DRL by interacting with a CFD-based environment (*FeniCs*) and is able to produce optimal 2D airfoil shapes at given flight conditions. Actions are taken by the DRL agent to move a finite number of points (between 2 and 4 points) to describe the airfoil shape. The reward is based on the achieved lift-to-drag ratio as well as the area of the shape described by the points. The research demonstrates that the agent is able to generate wing-like optimal shapes within 3000 episodes. The relatively low number of points that describe the airfoil as well as imposed restrictions on their positions in the 2D plane limits the generative capabilities of the detailed approach. Moreover, a single aerodynamic objective (lift-to-drag ratio) is investigated in this approach: it would be interesting to compare the learning abilities and generated airfoils of a DRL approach for different aerodynamic objectives.

In addition to being relevant to solving MDP-formulated problems, reinforcement learning-based optimization has been shown to outperform other traditional (Gradient Descent, Momentum, Conjugate Gradient, L-BFGS) and supervised methods, in terms of convergence speed and/or final objective value^35,36^. Hence, given the documented suitability of a RL-based approach to our problem, a custom RL environment for airfoil design is proposed.

In summary, previous studies have investigated the application of RL to aerodynamic shape design. However, they suffer from the following limitations, namely (i) limited exploratory freedom due to restricted action spaces, and (ii) learning is performed under a single aerodynamic objective. In the present research, these limitations are addressed by providing a continuous action space to the agent, increasing its overall generative freedom. Furthermore, by varying aerodynamic objectives, we demonstrate the robustness of the learning agent. The innovation of the present research resides in demonstrating that through an MDP approach to airfoil design, and provided a suitable DRL environment, an artificial agent is able to learn the optimal policy within a high dimensional design space, guiding the design of optimal airfoils under varying aerodynamic objectives.

## Problem definition

Airfoil shape optimization is a complex, non-linear and high-dimensional inverse problem relying on an iterative process to explore the design space. Current approaches have limitations. Gradient-based techniques lack robustness while gradient-free techniques are too computationally expensive when coupled with high-fidelity aerodynamic solvers; both are data-inefficient since do not leverage knowledge of previous experiments. Supervised machine learning approaches address some of these limitations but are constrained to the provided data. By representing the design of airfoils as a Markov decision process, a reinforcement learning methodology can be applied to address the limitations of past approaches. Specifically, by exploiting the capabilities of deep neural networks, DRL is able to (i) tackle non-linear problem of high-dimensionality while (ii) staying agnostic to details of the problem at hand. While DRL appears to be an innovative and promising approach to the airfoil shape optimization problem, the review of the literature presented in Section II highlights the following limitations: (i) a lack of exploratory freedom provided to the agent due to restricted action-spaces, and (ii) learning performed under limited aerodynamic objectives.

The present research brings forward a Markov decision process formulation of airfoil shape optimization, for which a Deep Reinforcement Learning approach appears most promising. The artificial agent trained through DRL learns the optimal policy that leads to best performing airfoils by directly modifying the airfoil shape. In order to fully leverage the exploratory capabilities of RL, considerable freedom is granted to the agent through an unrestricted action-space and large design space. Our approach allows the agent to successively modify the thickness and camber of the airfoil at selected chordwise positions, using continuous values. Hence, the only limitation in our approach is the potential non-convergence of highly unconventional shapes in the aerodynamic solver evaluating the agent-generated airfoils, which represents a physics-based limitation. Moreover, to demonstrate the learning abilities of the agent, the target aerodynamic metrics (lift-to-drag ratio, lift coefficient and drag coefficient), which serve as a basis for the reward, are varied as well as the starting shape that the agent initially observes. Different shape parametrizations are also explored, emphasizing the importance of shape parametrization for the given problem. The following sections discuss in more detail the DRL approach proposed and its implementation for the airfoil shape optimization problem.

## Approach

In the following section, we present the DRL environment that is developed to address the airfoil shape optimization problem as well as the artificial agent that is chosen. The built environment shares the main features of any reinforcement learning environment presented in Fig. 2. Here, an artificial agent interacts with an environment through three information signals (observation-action-reward) to create the most optimized airfoil shape at given flight conditions. Although describing airfoil design as an MDP provides some degree of formalism (requiring a states/actions/rewards approach), a DRL environment cannot simply be used as a plug-and-play or black-box tool. Rather, the DRL environment must be tailored to best fit this particular design problem. As such, this research investigates various action spaces and shape parametrizations possible, and results are presented using a custom DRL environment.

### Agent and environment

**Figure 3:**
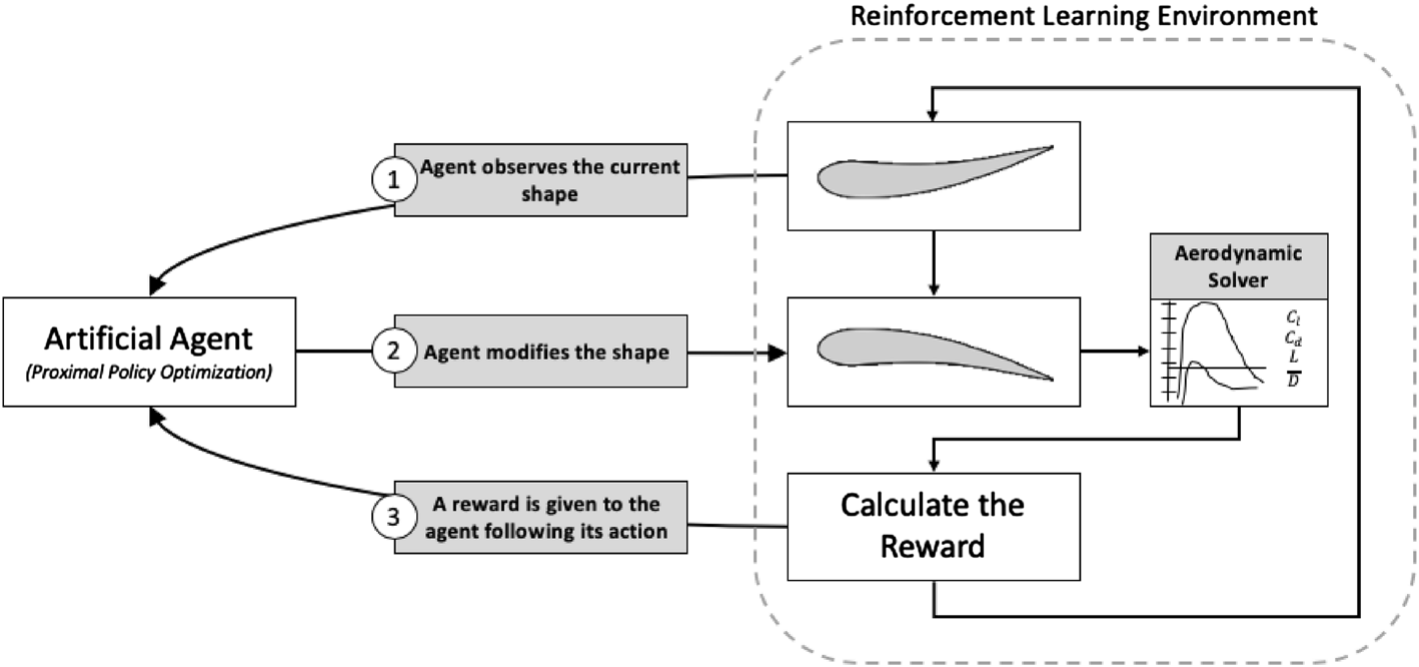
Custom RL environment for Airfoil Shape Optimization.

The agent starts by observing an airfoil shape represented by a finite number of points: this constitutes the current state of the environment. The agent then chooses to perform an action on this environment by modifying the airfoil shape. Following this action, the modified shape is run in a low-fidelity aerodynamic solver (*Xfoil*) to evaluate the performance of the airfoil shape, such as the the lift coefficient *C*_*l*_, the drag coefficient *C*_*d*_ and lift-to-drag ratio *L*/*D*. These performance metrics serve as a basis for the reward: if the lift-to-drag ratio increases compared to the previous unmodified shape, the agent receives a positive reward, signaling the agent of its positive behavior, and receives a negative reward if this ratio decreases, punishing the agent for its poor behavior. This observation/action/reward cycle is repeated for a given number of iterations forming an episode. At the end of an episode, a general score can be calculated to estimate the agent’s overall performance, based on the accrued performance metrics of the modified airfoils at each cycle (sum of the *L*/*D* at each cycle for example). The agent’s interactions with the environment over one cycle are depicted in Fig. 3.

Holistic airfoil aerodynamic metrics were used as the basis for the reward (such as lift coefficient *C*_*l*_, the drag coefficient *C*_*d*_ and lift-to-drag ratio *L*/*D*) instead of local flow solutions (such as *C*_*p*_), because (i) local flow conditions tend to be solver-specific, hindering the generalization capabilities of the proposed approach, and (ii) the solver used here (*XFOIL*) does not easily output such local conditions. Hence, to ensure sufficient generalization of the proposed approach, and to simplify the development of the RL environment, holistic airfoil aerodynamic metrics were chosen over local flow conditions to calculate the reward. Training of the RL agent is however specific to given flow conditions.

Using the proposed environment and under fixed Mach, Reynolds and angle of attack conditions, three exploratory tasks were carried out to demonstrate the learning ability of the agent:

- *Task 1*: Starting with a symmetric airfoil (NACA0012) having $L/D = 0$, the agent must (i) modify the airfoil shape to achieve highest *L*/*D*, (ii) modify the airfoil shape to achieve highest *C*_*l*_, (iii) modify the airfoil shape to achieve highest endurance $C^{3/2}_{l}/C_{d}$, and (iv) modify the airfoil shape to achieve lowest *C*_*d*_.
- *Task 2*: Starting with a high-performance airfoil (having high *L*/*D*), the agent must modify the airfoil shape to achieve highest *L*/*D*.
- *Task 3*: Starting with a low-performance airfoil (having low *L*/*D*) the agent must modify the airfoil shape to regain high *L*/*D*.

### Action space and reward

The observation provided by the environment to the agent is a n-set of points describing the airfoil shape. Different approaches regarding the action space were considered.

**Figure 4:**
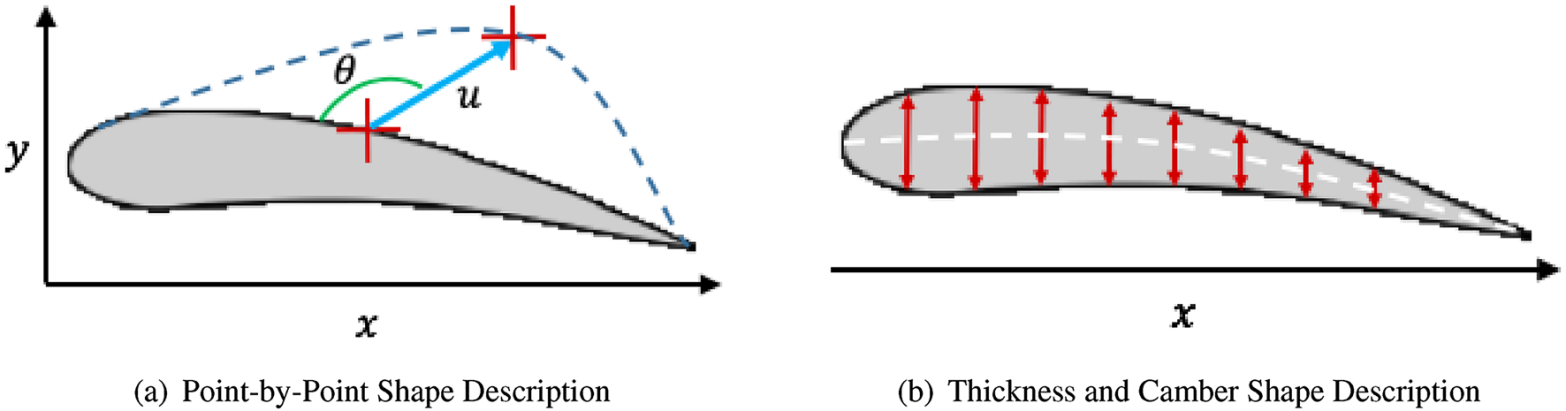
Two Explored Action Spaces.

A first point-by-point approach was considered. As illustrated in Fig. 4a, the agent can select any point individually and move it following a 3-set of actions: (i) choice of a point within the (*x*, *y*) plane, (ii) choice of a translation amount *u*, and (iii) choice of a direction of modification $\theta$. The main issues encountered with this approach is that it can lead to problematic modified shapes. For instance, a point located on the extrados could be moved in such a way that after modification it would be located under the intrados, thus leading to a “crossed” or tangled shape. Imposing limitations on the movements of the points results in added complexity as well as decreases the generalization capability of our overall approach. This has led us to consider a second approach.

In a second approach illustrated in Fig. 4b, the airfoil shape is described by a set of *x* coordinates at which are associated local thickness and camber. The agent is given a 3-set of actions: (i) choice of a specific *x* position along the airfoil’s chord line, (ii) a change in thickness at the selected *x* position, and (iii) a change in camber at the selected *x* position. This airfoil shape description and associated action space leads to an increase in the number of realistic airfoils produced.

The immediate reward *r* is based on the change in the performance metric of interest associated to a given shape. More precisely, we define the reward as the difference in the metric between two successive shape changes, for example changes in *L*/*D*:

10 $$\begin{aligned} r = L/D_{current} - L/D_{previous} \end{aligned}$$

If the change in the shape leads to an increase in *L*/*D* or *C*_*l*_ compared to the previous shape, the agent receives a positive reward. When the metric of interest is *C*_*d*_, the reward is based on a decrease in the drag coefficient.

In the event where the aerodynamic solver (i.e., *XFOIL*) does not converge due to too abrupt geometric changes brought to the airfoil, the agent receive a very negative reward (differing by an order of magnitude when compared to the above reward), punishing the agent for its poor decision. As a result, geometric abnormality constraints are imposed through the convergence (or non-convergence) of the aerodynamic solver. Other, more complex, geometric abnormality constraints for airfoil shape optimization have however been implemented. In^37^, a GAN-based abnormality scoring method is devised and used to filter out unrealistic airfoil shape, improving convergence and increasing the efficiency of the aerodynamic shape optimization problem at hand.

### Airfoil shape description

**Figure 5:**
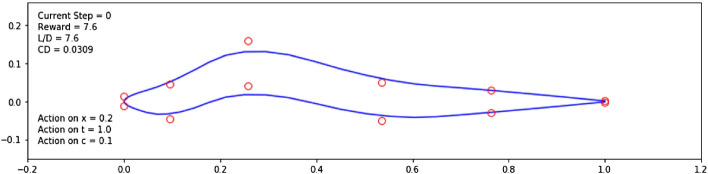
Abrupt Shape Changes Performed by the Agent During Exploration.

The 2D airfoil shape that the artificial agent observes and seeks to optimize is represented by a finite set of control points in the (*x*, *y*) plane. However, transforming this set of control points into a representative airfoil that can be run in an aerodynamic solver calls for a shape parametrization, which is a critical step in any aerodynamic shape optimization process^38^. More specifically, in our given problem, an agent is tasked with bringing certain modifications to the control points describing the airfoil shape to increase the airfoil’s performance. Inherent to the learning process, a first exploratory phase happens during which the agent can take actions leading to points being moved to positions from which the resulting shape is unusual, even non airfoil-like (see Fig. 5).

A smoothing process by which a curve that tries to best fit the control points is used to represent the shape resulting from the control points available to the agent. A wide range of airfoil parametrization approaches are explored and compared in^2,38^. It is shown that many of these approaches are equivalent to a particular class of parametrization, namely Béziers curves and B-Splines. Previous work has demonstrated the advantages of this class of parametrization towards airfoil representation. In^39^, it is shown that Béziers splines only require few variables, hence making them a candidate for efficient airfoil definition. However, only limited local control of the curve can be achieved, as bringing a modification to any point defining a Béziers spline modifies the curve as a whole^2^. It is demonstrated in^40^ that more complex airfoil definitions can be achieved through B-Splines, which allow for local changes when modifying a single point. As a result, B-Splines exhibit noteworthy advantages for our research over other parametrizations: (i) they allow for highly localized shape changes, (ii) the increase in the number of control points does not increase the degree of the curves, and (iii) they offer more flexibility, hence can more accurately fit a desired geometry while remaining numerically stable. In our present research, three parametrization approaches were investigated, differing in the number of control points and number of splines used to describe the shape.

#### First parametrization

**Figure 6:**
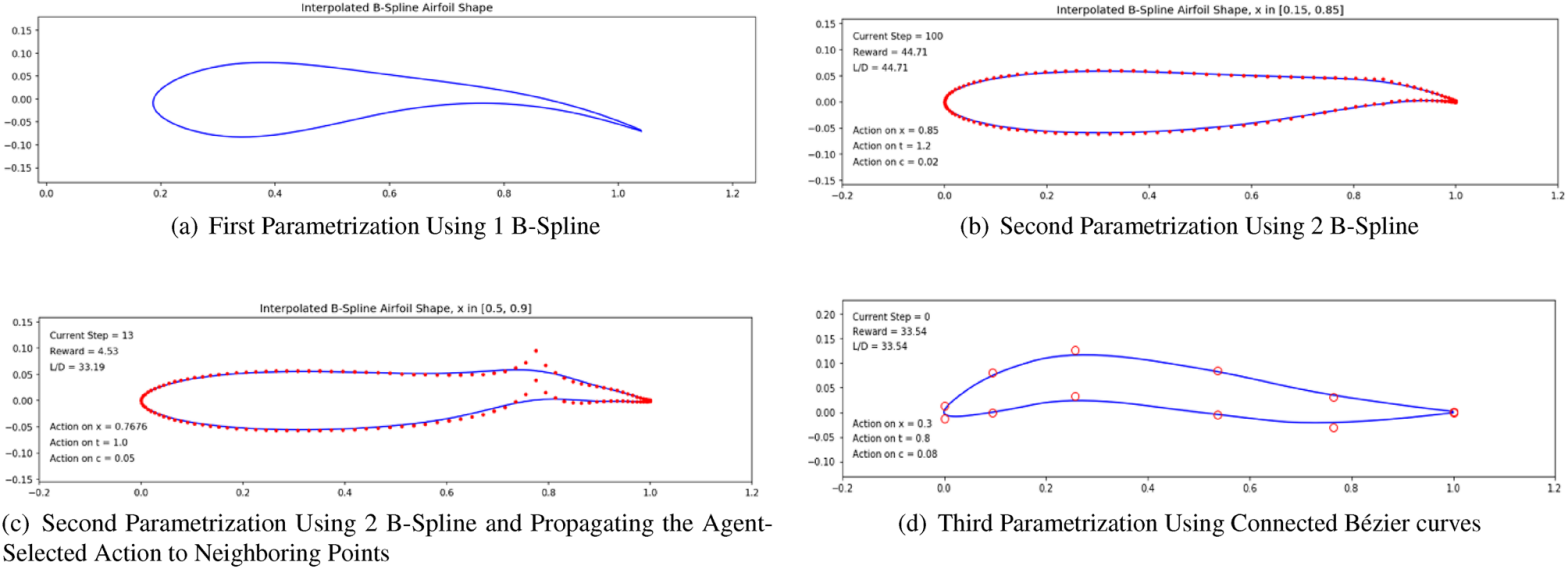
Investigated approaches for the airfoil shape description.

In *Task 1* of our research, each episode starts with a symmetric NACA0012 airfoil. The set of points describing this airfoil was taken from the *UIUC Airfoil Coordinates Database*^41^. This particular airfoil counts 131 points. The resulting airfoil shape is given by a single B-Spline parametrized by these 131 points, which can then be run in an aerodynamic solver (*Xfoil* here). When the agent modifies the airfoil by moving control points, the B-Spline representation of these points changes accordingly. The main issue observed when using a single B-Spline is that there is no guarantee that the leading and trailing edges of the airfoil shape will stay at fixed positions. This stems from the fact that the B-Spline is only guaranteed to go through two anchor points, which are the first and last points describing the shape i.e., the two most extreme points at the trailing edge, as illustrated in Fig. 6a. Keeping the leading and trailing edges at fixed positions ($(x=0, y=0)$ and $(x=1, y=0)$, respectively) is needed to ensure that we are comparing airfoils at a fixed angle of attack ($\alpha =0$ in this case). To address this, we chose to investigate another parametrization based on two B-Splines.

#### Second parametrization

Using two B-Splines each describing the extrados and intrados of the airfoil (Fig. 6b), we are able to keep the leading and trailing edges at fixed positions. This ensures that the performance of the produced airfoils can be compared since all are at constant angle of attack. However, another issue was identified in this second approach that originates from the large number (131 points) of control points used. Due to the large number of control points, when the agent chooses to modify the thickness and camber at a selected *x* coordinate, because of the weight of the unchanged neighboring points, the modification poorly reflects on the resulting B-Spline shape. To put it in simpler terms, the action of the agent on the control points is not reflected on the B-Spline representing the airfoil shape. One way to address this issue is to propagate the agent’s action to neighboring points, as illustrated in Fig. 6c. However, this has a major drawback on the agent’s learning: propagating the action masks the direct impact of the agent’s selected action.

#### Third parametrization

To address both of the issues observed previously, we chose to describe the shape using a reduced number of points of approximately 12 control points. The consequence of this is that the agent has a reduced number of points from which to choose, thus decreasing the degree of freedom and dimension of the action space, but makes the impact of the agent’s action more interpretable by the agent, leading to enhanced learning. In this third parametrization, we use composite Bézier curves, which are a series of connected quadratic Bézier curves defined by the control points^42^. These curves allow for the resulting shape to be sufficiently smooth, thus increasing the convergence rate of the aerodynamic solver, even in cases where the agent’s action leads to unusual shapes. The 12 control points are extracted from the previous 131, originating from the *UIUC* NACA0012 file^41^ and selected to be evenly spaced. Two close points are selected to describe the rounded leading edge and sharp trailing edge. This third parametrization was selected moving forward and is illustrated in Fig. 6d.

### Emulator–aerodynamic solver

For the agent to evaluate the impact of its action on the airfoil, the modified shape must be run in an aerodynamic solver to obtain the aerodynamic properties of the modified airfoil shape. In the present research, we chose *Xfoil* as the aerodynamic solver. *Xfoil* is an interactive program developed at *MIT* for the analysis of subsonic 2D airfoils^43^. It handles both viscid and inviscid flows and provides lift, drag, pitching moments and other aerodynamic parameters associated with a given airfoil. The choice of *Xfoil* over other solvers is guided by its relatively low-fidelity allowing for quick run times. In turn, this allows the agent to maximize its interaction with the environment by testing airfoil shape modifications quickly. However, *Xfoil* exhibits some limitations for our present study: (i) poor convergence for unconventional airfoil designs, and (ii) convergence is history-dependent and sensitive to the initial shape. The former leads to very low convergence rates during the agent’s initial learning phase, where exploration is high and the agent produces highly unusual airfoil shapes. The latter is mitigated by running the solver on a series of gradually modified shapes between two successive agent actions.

### Selecting the DRL agent

As mentioned, deep reinforcement learning trains an artificial neural network (ANN) to learn the optimized behavior within a given environment by interacting with the environment through a set of observation/action/reward signals. The ANN or DRL agent are used interchangeably hereafter. Different DRL algorithms have been developed over time. Revealing the power of DRL, Deep Q Networks (DQN) have been applied to the classic *Atari 2600* games and have achieved human-level performance at these games^33^. DQN has then been perfected into Double Deep Q Networks (DDQN), achieving even higher performance^44^. Both DQN and DDQN are *value-based* methods and apply to discrete action spaces^33^. *Policy-based* methods, such as the deterministic policy gradient (DPG) algorithms^45^ and deep DPG (DDPG)^46^ have been developed in parallel. Being *policy-based* methods, DPG and DDPG can address both continuous action and state spaces and exhibit better stability and convergence properties compared to DQN^1^. Finally, Proximal Policy Optimization (PPO^47^) has demonstrated higher efficiencies in the policy gradient algorithm class.

In our present research, the action space is composed of three actions: (i) choice of $x\in [0, 1]$, (ii) choice of a continuous value by which to change the local thickness, and (iii) choice of a continuous value by which to change the local camber. Hence, we chose to use the PPO method for the learning of our DRL agent for its ability to handle continuous action spaces, but also because it exhibits quicker learning speeds than other policy gradient methods and is less complex mathematically^1^.

### Deep reinforcement learning framework

Now that we have formalized the observation, action and reward for our given problem, we need to provide the selected DRL agent with a custom reinforcement learning environment. This environment is similar to video game-based environments such as the classic *Atari 2600* widely used to test and benchmark DRL agents^33^. In our case, the DRL agent observes a set of control points defining an airfoil shape instead of a video game screen and can choose to modify the thickness and camber at a selected chordwise position in the same way that it would take actions on the joysticks of the video game. The modified shape is then run in *Xfoil* to get the associated performance metrics, which serve as a basis for the reward attributed for the taken action. The given reward is comparable to a change in the game score in video game-based environments.

We chose to develop this custom reinforcement learning environment in *Python* for several reasons. First, *Python* is currently an extremely widespread programming language, having an extensive community providing high-quality documentation. It is also a high-level object oriented language, making it accessible for rapid engineering and scientific applications. Secondly, numerous machine learning packages are distributed in *Python* and make it the most represented language in the machine learning community. For example, *TensorFlow*, an open-source software developed at *Google Brain*, has a *Python* library that provides users with a range of machine learning tools with a focus on the implementation and training of deep neural networks^48^. Finally, *Python* was selected for the ease to integrate *Xfoil* into the reinforcement learning environment.

Several toolkits for building a custom reinforcement learning environment exist. *Gym*, developed by *OpenAI*, is a *Python*-based open-source toolkit that provides an abstraction for a custom environment around a common interface, defined by four core functions^49^:

- *Initialization*: initialize the environment and define the observation and action spaces.
- A *Step* function: given an action chosen by the agent, it dictates how the environment is modified and gives the associated reward.
- A *Reset* function: resets the environment when the end of an episode is reached.
- A *Render* function: provides visualizations of the environment. These visualization can come in the form of numerical values keeping track of the observation/action/reward set or a visual representation of the environment (such as the screen of the video game).

It is important to understand that *Gym* provides a template for the environment, not the DRL agent^49^. The DRL agent (here chosen as the PPO algorithm) will have to interact with the *Gym* environment through the observation/action/reward signals sent from and to the environment itself, as illustrated in Fig. 3.

Multiple libraries implementing RL and DRL algorithms have been developed. In this research, we choose the *Stable Baselines* library (created at Inria/ENSTA ParisTech) for our DRL agent^50^. This choice was motivated by *Stable Baselines’* (i) ease to integrate with *Gym*-based custom environments, (ii) state of the art RL methods, including PPO, and (iii) a well-developed documentation and community.

To summarize, a custom RL environment was developed for the airfoil shape optimization problem, along the guidelines of a *Gym* environment, and an off-the-shelf implementation of the PPO agent was used to learn the optimal policy within our provided environment.

## Results

In the following section, we present the learning capabilities of the DRL agent with respect to optimizing an airfoil shape, trained in our custom RL environment. Different objectives for the DRL agent were tested, gathered into three tasks. In *Task 1*, the environment is initialized with a symmetric NACA0012 airfoil and successive tests were performed in which the agent must (i) maximize the lift-to-drag ratio *L*/*D*, (ii) maximize the lift coefficient *C*_*l*_, (iii) maximize endurance $C^{3/2}_{l}/C_{d}$, and (iv) minimize the drag coefficient *C*_*d*_. In *Task 2*, the environment is initialized with a high performing airfoil having high lift-to-drag ratio and the agent must maximize this ratio. The goal is to test if the learning process is sensitive to the initial state of the environment and if higher performing airfoils can potentially be produced by the agent. In *Task 3*, the environment is initialized with this same higher performing airfoil, but has been flipped along the *y* axis. Under this scenario, we investigate the impact of initializing the environment with a poor performing airfoil on the agent and determine if the agent is able to modify the airfoil shape to recoup a high lift-to-drag ratio. Overall, these tasks demonstrate the learning capabilities of the DRL agent to meet specified aerodynamic objectives.

### Flow conditions

Since we are interested in evaluating the drag of the agent-produced airfoils, the viscous mode of *Xfoil* is used. In viscous flow conditions, *Xfoil* only requires the user to specify a Reynolds number (*Re*) and an airfoil angle of attack $\alpha$. In all tasks, the flow conditions specified in *Xfoil* were kept constant. A zero-degree angle of attack and Reynolds number equal to 10^6^ were selected to define the design point for the flow conditions. The decision to keep the airfoil’s angle of attack at a fixed position is motivated by the interpretability of the agent’s policy. A less constrained problem, in which the agent can modify the angle of attack, would significantly increase the design space, leading to less interpretability of the agent’s actions. Additionally, the angle of attack is chosen to be fixed at zero in order to easily compare the performance of agent-generated shapes with those found in the literature. The Reynolds number was chosen to represent an airfoil shape optimization problem at speeds under the transonic flow regime^15^. Hence, given the relatively low *Re* number chosen, the flow is incompressible over the airfoil, although *Xfoil* does include some compressibility corrections when approaching transonic regimes (Karman-Tsien compressibility correction,^43^). All airfoils are thus compared at zero angle of attack.

### DRL agent parameters

Two parameters relating to the PPO algorithm in *Stable Baselines* can be set, namely the discount factor $\gamma$ and the learning rate. The discount factor impacts how important future rewards are to the current state: $\gamma = 0$ will favor short-term reward whereas $\gamma = 1$ aims at maximizing the cumulative reward in the long run. The learning rate controls the amount of change brought to the model: it is a hyperparameter tuning the PPO neural network. For the PPO agent, the learning rate must be withing $[5\times 10^{-6}, 0.003]$. A study of the effects of the discount factor and learning rate on the learning process was conducted. This study shows that optimal results are found when using a discount factor $\gamma = 0.99$ and learning rate equal to 0.00025.

### Environment parameters

In building our custom environment, we have set some parameters to limit the generation of unrealistic shapes by the agent. These parameters help take into account structural considerations as well as limit the size of the action space. For instance, we define limits to the thickness of the produced shape. If the generated shape (resulting from the splines represented by the control points) exhibits a thickness over or under a specified limit value, the agent will receive a poor reward. Regarding the action space, we set bounds for the change in thickness and camber. This allows the agent to search in a restricted action space thus eliminating a great number of unconverged shapes resulting from actions bringing changes to the airfoil shape that are too extreme. These parameters are given in Table 2. Moreover, the *iterations* parameter is the number of times *Xfoil* is allowed to rerun a calculation for a given airfoil in the event the solver does not converge. Having a high *iterations* number increases the convergence rate of *Xfoil* but also increases run times.

**Table 2:** Environment parameters.

Minimum allowable change in local thickness	90%
Maximum allowable change in local thickness	110%
Lower bound for local change in camber	− 0.005
Upper bound for local change in camber	0.005
Minimum allowed thickness (over whole shape)	0.03
Maximum allowed thickness (over whole shape)	0.15

### Task 1

**Figure 7:**
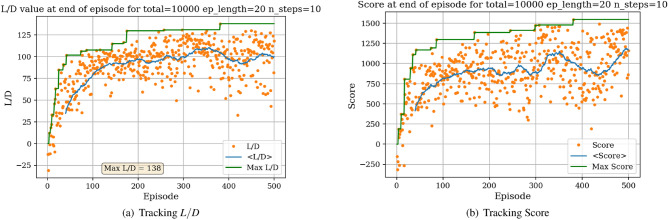
Learning curves for max *L*/*D* objective starting with a symmetric airfoil.

The environment is initialized with a symmetric airfoil having $L/D = 0$, $C_{l} = 0$ and $C_{d} = 0.0054$ at $\alpha = 0$ and $Re = 10^{6}$. In a first experiment, the agent is tasked with producing the highest lift-to-drag airfoil, starting from the symmetric airfoil. During each experiment, the agent is trained over a total number of iterations (defined as the *total timestep* parameter), which are broken down into episodes having a given length (defined as the *episode length* parameter). The DRL agent is updated (i.e., changes are brought to the neural network parameters) every *N steps*. At the end of an experiment, several results are produced. Figure 7a displays the *L*/*D* of the airfoil successively modified by the agent at the end of each episode.

In Fig. 7a, each dot represents the *L*/*D* value of the shape at the end of an episode and the blue line represents the *L*/*D* running average value over 40 successive episodes. The maximum *L*/*D* obtained over all episodes is also displayed. Settings regarding the total number of iterations, episode length and N steps for the experiment are given above the graph. It can be observed from Fig. 7a that starting with a low *L*/*D* during early episodes, the *L*/*D* at the end of an episode increases with the number of episodes. Though significant variance in the *L*/*D* at the end of an episode can be seen, with values ranging between $L/D = -30$ and $L/D = 138$, the average value however increases and stabilizes around $L/D = 100$. This increase in *L*/*D* suggests that the agent in able to learn the appropriate modifications to bring to the symmetric airfoil resulting in an airfoil having high lift-to-drag ratio. We are also interested in tracking a score over a whole episode. Here, we arbitrarily define this score as the sum of the *L*/*D* of each shape produced during an episode. For instance, if an episode is comprised of 20 iterations, the agent will have the opportunity to modify the shape 20 times thus resulting in 20 *L*/*D* values. Summing these values corresponds to the score over one episode. If the agent produces a shape that does not converge in the aerodynamic solver, a value of 0 is added to the score, thus penalizing the score over the episode if the agent produces highly unrealistic shapes. The evolution of the score with the number of episodes played is displayed in Fig. 7b.

**Figure 8:**
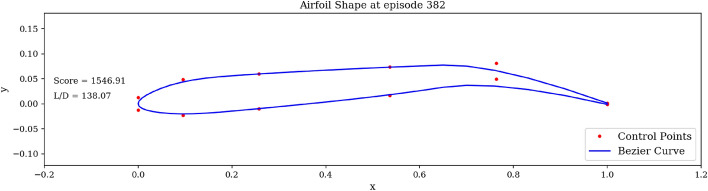
Agent-produced airfoil shape having highest *L*/*D* over training.

Figure 7b shows the significant increase in the average score at end of episode, signaling that the agent is learning the optimal shape modifications. We can then visualize the best produced shape over the training phase in Fig. 8.

**Figure 9:**
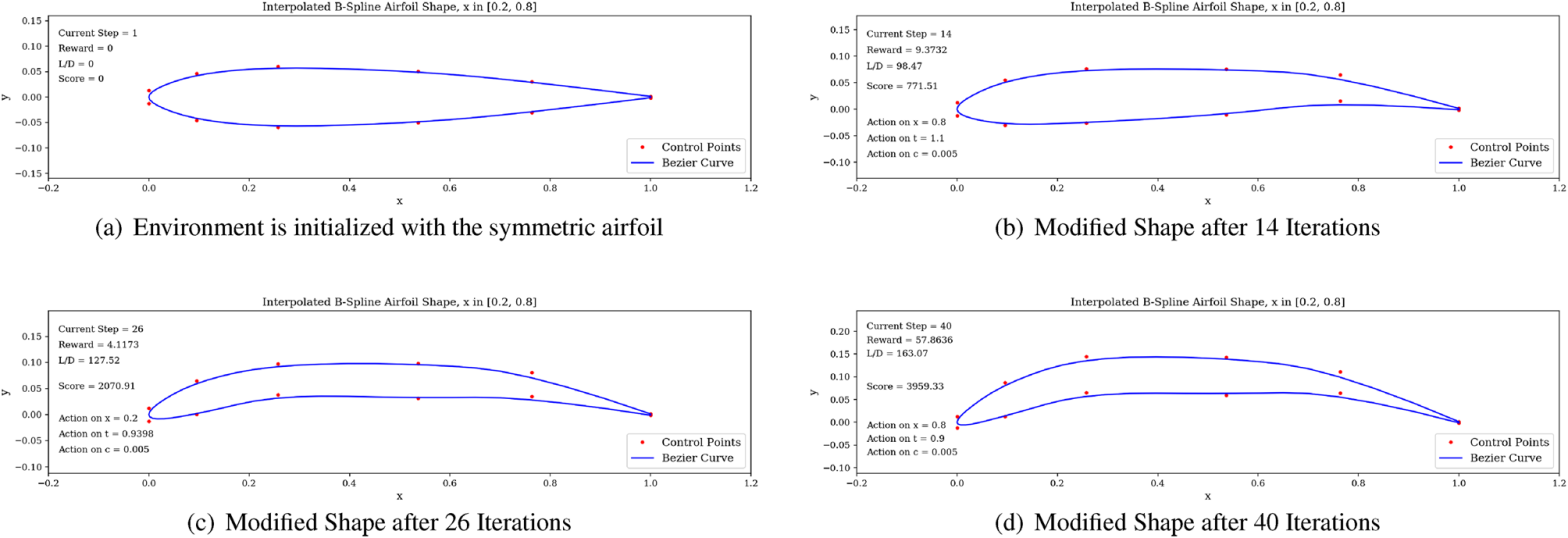
Trained agent modifies shape to produce high *L*/*D*.

In Fig. 8, the red dots are the control points accessible to the agent. The blue curve describing the shape is the spline resulting from these control points. It is interesting to observe that the optimal shape produced shares the characteristics of high lift-to-drag ratio airfoils, such as those found on gliders, having high camber and a drooped trailing edge. Finally, we run the trained agent on the environment over one episode and observe the generated shapes in Fig. 9. Starting from the symmetric airfoil, we can notice the clear set of actions taken by the agent to modify the shape to increase *L*/*D*. The experiment detailed above was repeated by varying total timesteps, episode lengths and N steps.

**Figure 10:**
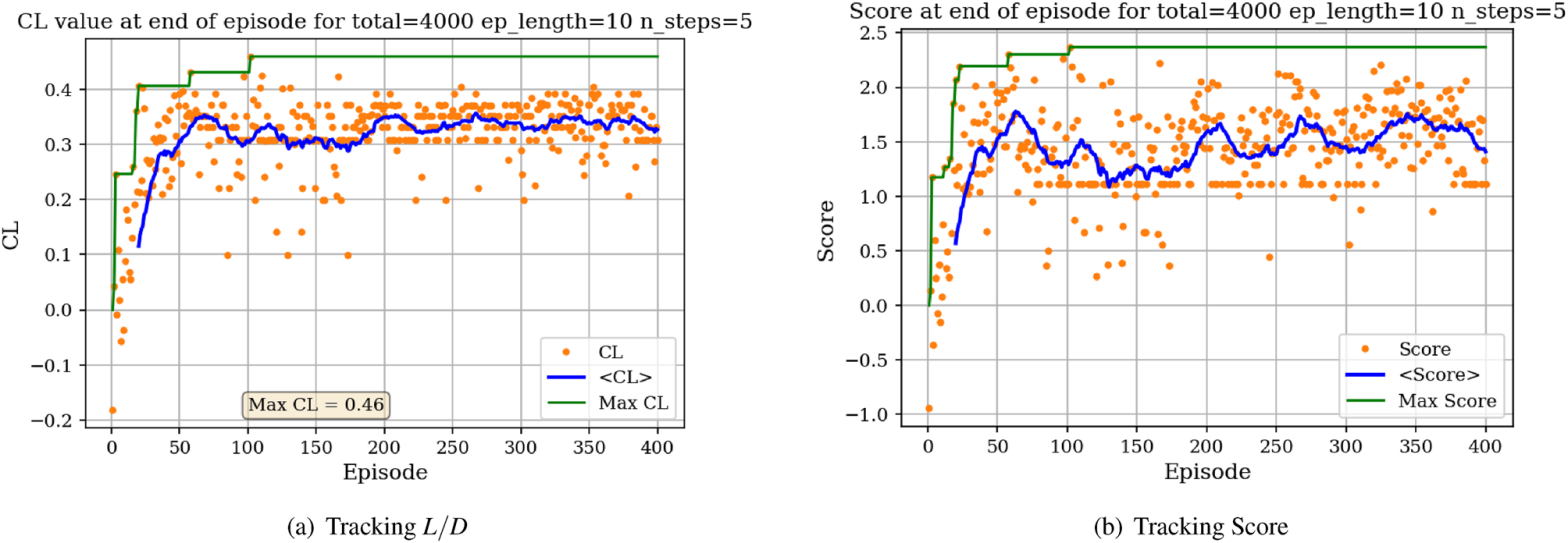
Learning curves for max *C*_*l*_ objective starting with a symmetric airfoil.

**Figure 11:**
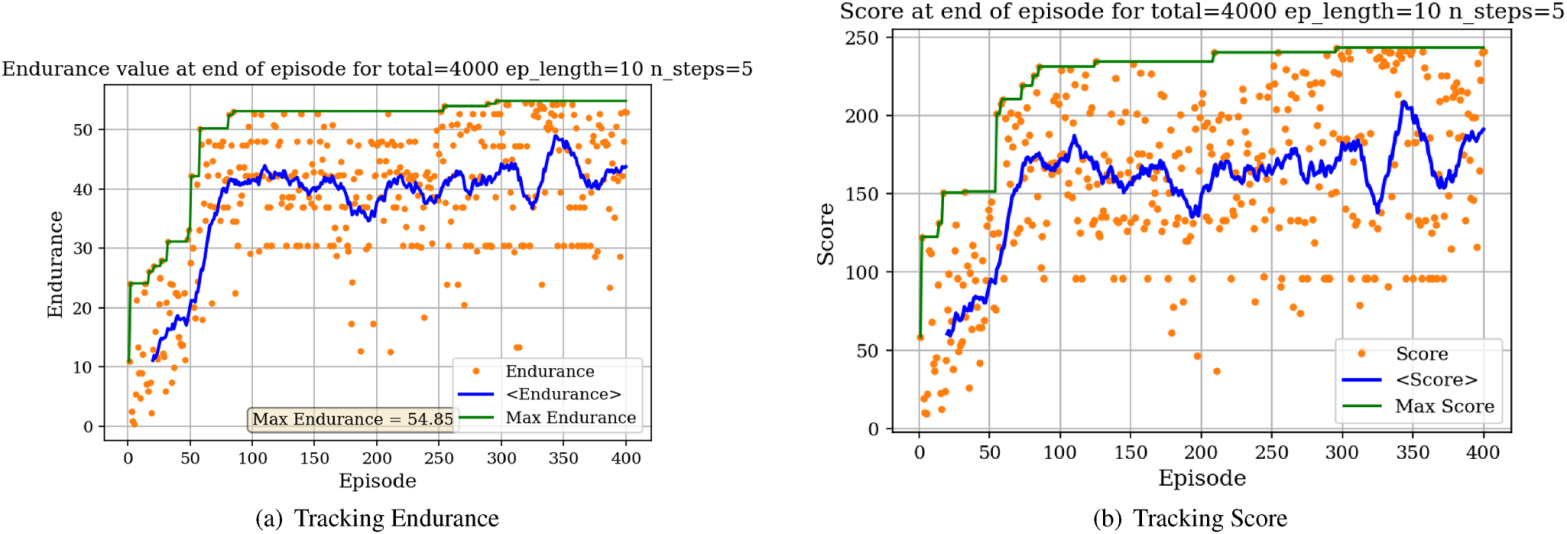
Learning curves for max $C^{3/2}_{l}/C_{d}$ objective starting with a symmetric airfoil.

**Figure 12:**
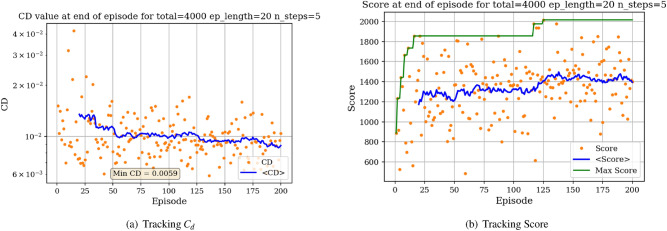
Learning curves for min *C*_*d*_ objective starting with a symmetric airfoil.

**Figure 13:**
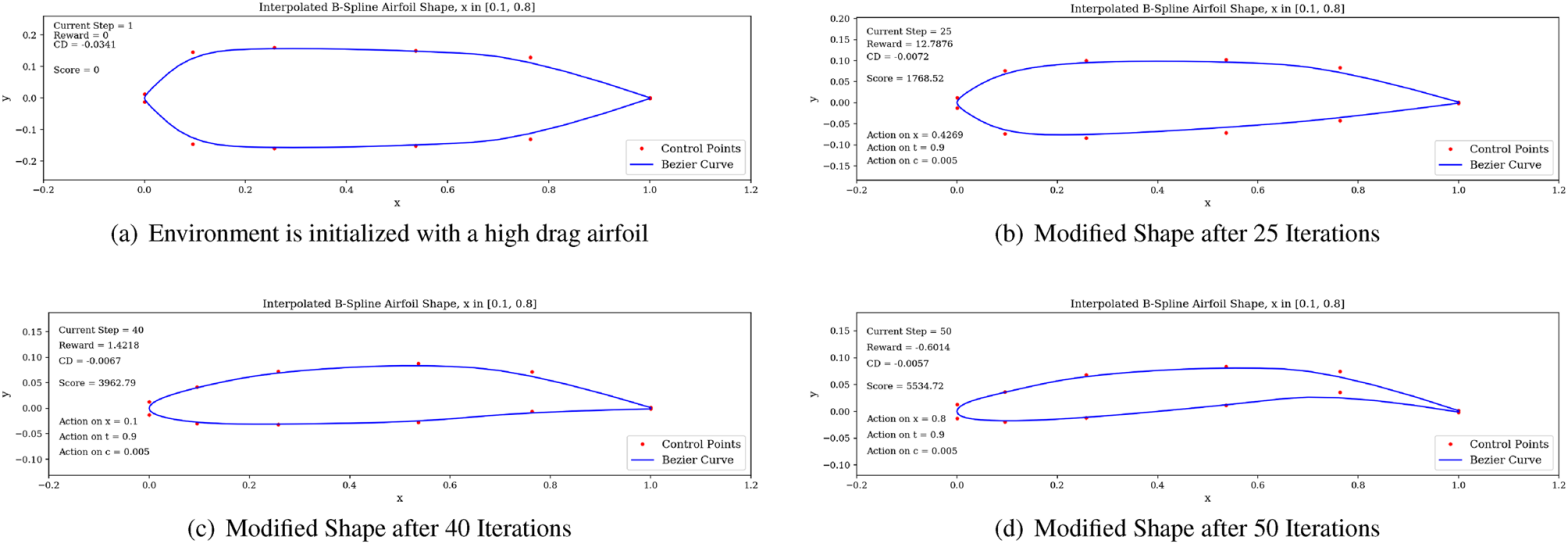
Trained agent modifies shape to produce low *C*_*d*_ starting with a low-performance airfoil.

We then proceed to train the agent under different objectives: maximize *C*_*l*_, maximize endurance and minimize *C*_*d*_. Associated learning curves and modified shapes can be found in Figures 10, 11, 12, 13.

For the minimization of *C*_*d*_ objective, the environment is initialized with a symmetric airfoil having *C*_*d*_ = 0.0341. This change in initial airfoil, compared to the previously used NACA0012 is justified by enhanced learning visualizations.

Similarly, the results show a clear learning curve during which both the metric of interest and the score at end of episode increase with the number of episodes. The learning process appears to happen within the first 100 episodes as signaled by the rapid increase in the score and then plateaus, oscillating around an average score value.

### Task 2

**Figure 14:**
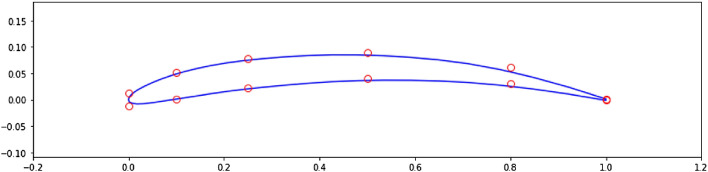
Eppler 58 high lift-to-drag ratio airfoil.

**Figure 15:**
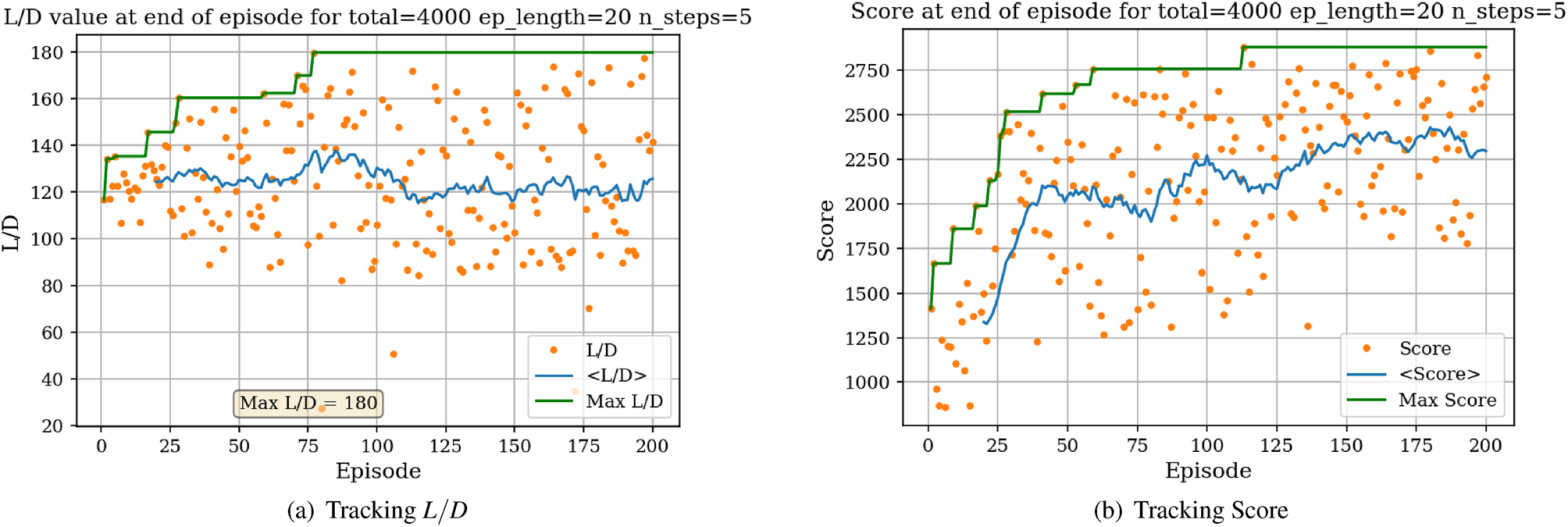
Learning curves for max *L*/*D* objective starting with a high *L*/*D* airfoil.

A second set of experiments was performed to assess the impact of the initial shape. The environment is initialized with a high performing airfoil (i.e., having a relatively high lift-to-drag ratio) and the agent is tasked with bringing further improvement to this airfoil. We chose this airfoil by investigating the *UIUC* database^41^ and selected the airfoil having the highest *L*/*D*. This corresponds to the Eppler 58 airfoil (e58-il) having $L/D = 160$ at $\alpha = 0$ and $Re = 10^{6}$, displayed in Fig. 14. Results for this experiments are displayed in Fig. 15.

It is interesting to compare the learning curves and average scores achieved when starting with the symmetric airfoil and the high performance airfoil.

**Figure 16:**
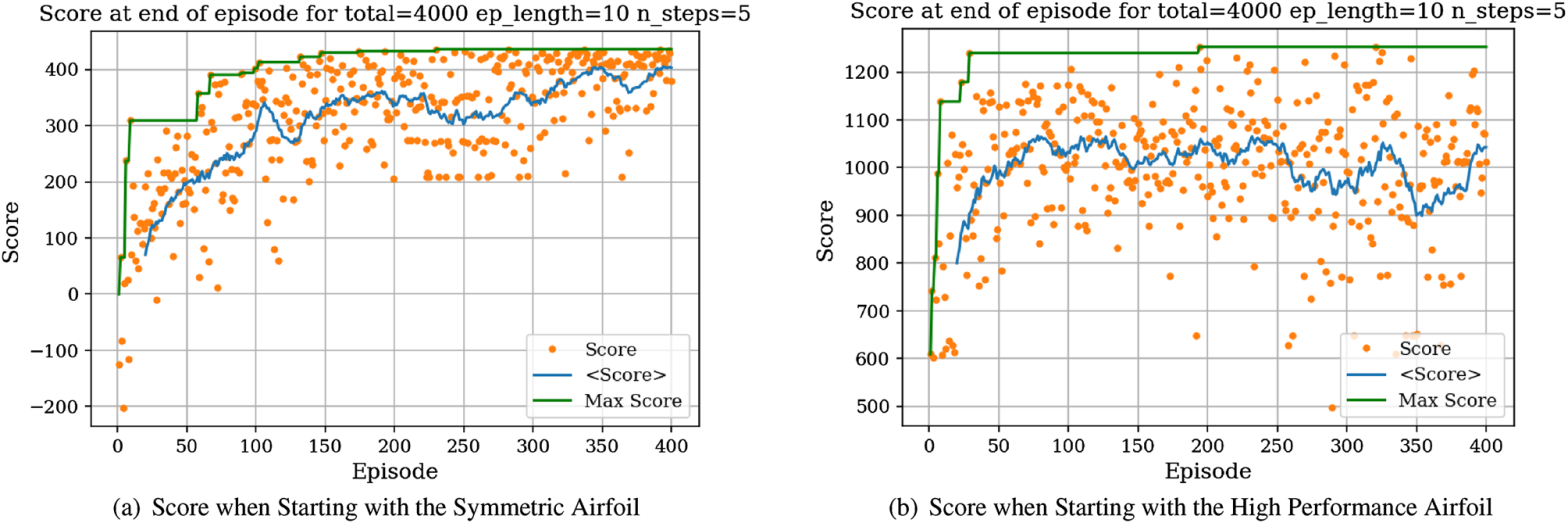
Initial airfoil impact on the learning curve.

In Fig. 16, we can observe that for both initial situations there is an increase in the average score during early episodes followed by stagnation, demonstrating the learning capabilities of the agent. However, the plateaued average score reached is significantly higher when the environment is initialized with the high performance airfoil, given that the environment is initialized in an already high-reward region (through the high-performance airfoil). Additionally, it was observed that a slightly higher maximum *L*/*D* value could be achieved when starting with the high lift-to-drag ratio airfoil. Overall, Task 1 and Task 2 emphasize the robustness of the RL agent to successfully converge on high *L*/*D* airfoils, regardless of the initial shapes (in both experiments, the agent converges on airfoils having $L/D > 160$). The agent-generated airfoil for *Task 2* is represented in Fig. 21a.

### Task 3

For *Task 3*, the starting airfoil is a version of the Eppler 58 airfoil that has been flipped around the *y* axis. As such, the starting airfoil has a lift-to-drag ratio opposite of the Eppler 58 (i.e., $L/D = -160$), thus exhibits low aerodynamic performance. The goal for this task is for the agent to modify the shape into a high performing airfoil, having high *L*/*D*.

**Figure 17:**
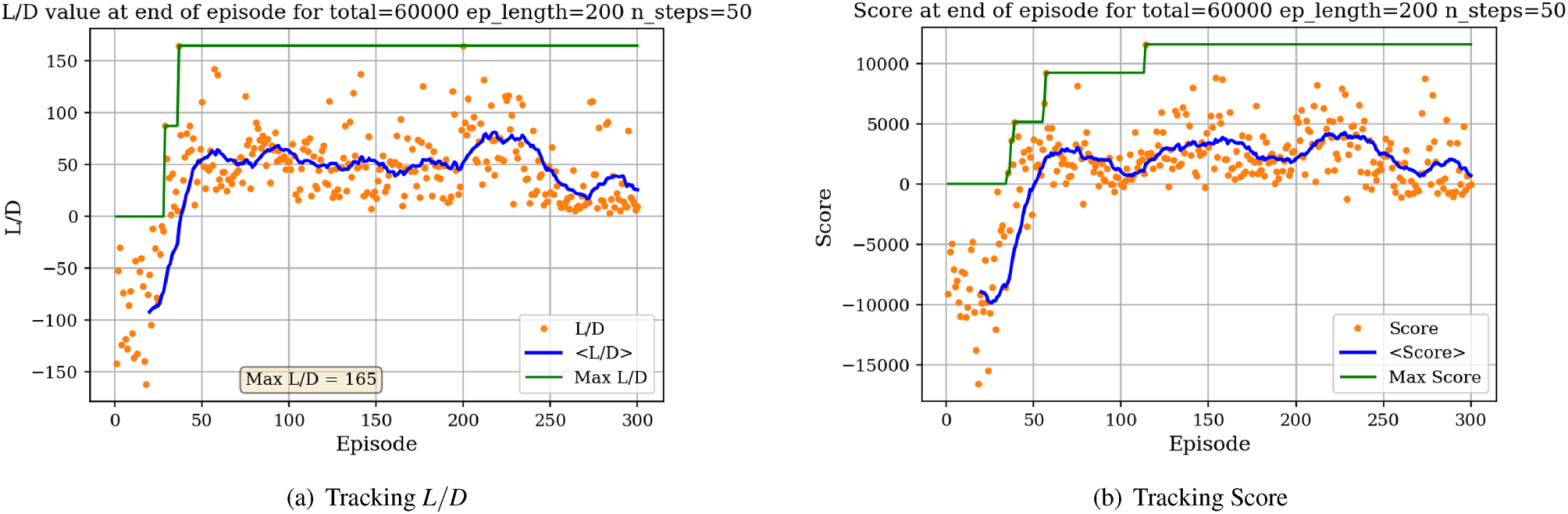
Score and *L*/*D* learning curves when starting with a low performance airfoil.

In Fig. 17, we display the learning curves associated to the score and *L*/*D* value at the end of each episode when the environment is initialized with the flipped e58 airfoil at the beginning of each episode. A noticeable increase in both the score and *L*/*D* values between episode 30 and episode 75 can be observed, followed by a plateau region. This demonstrates that the agent is able to learn the optimal policy to transform the poor performing airfoil into a high performing airfoil by bringing adequate changes to the airfoil shape. The agent then applies this learned optimal policy after episode 100. Moreover, the agent is capable of producing airfoils having lift-to-drag ratios equivalent or higher than the Eppler e58 high-performance airfoil, signaling that the initial airfoil observed by the agent does not impact the optimal policy learned by the agent, but rather only delays its discovery (see Figs. 15 and 17).

**Figure 18:**
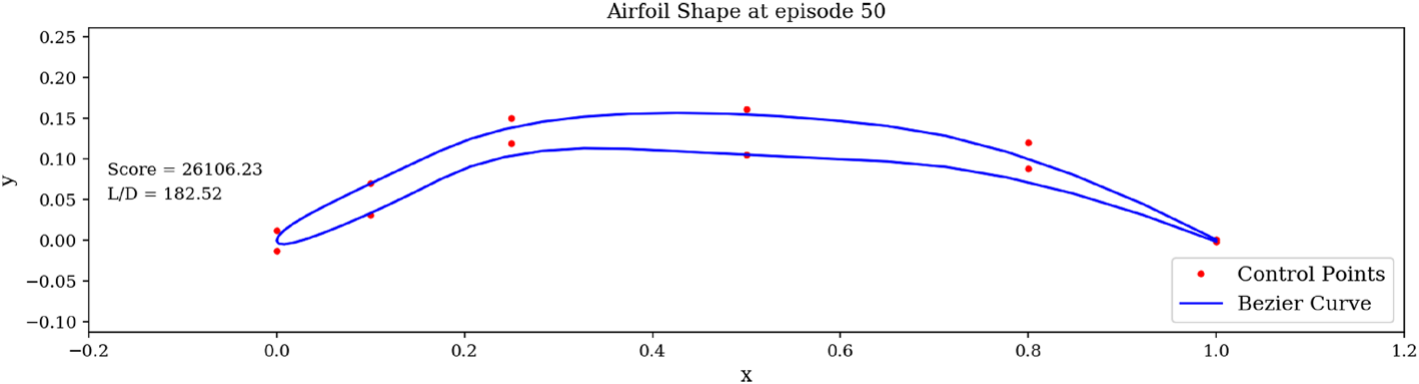
Agent-produced airfoil shape when starting with low performance airfoil.

**Figure 19:**
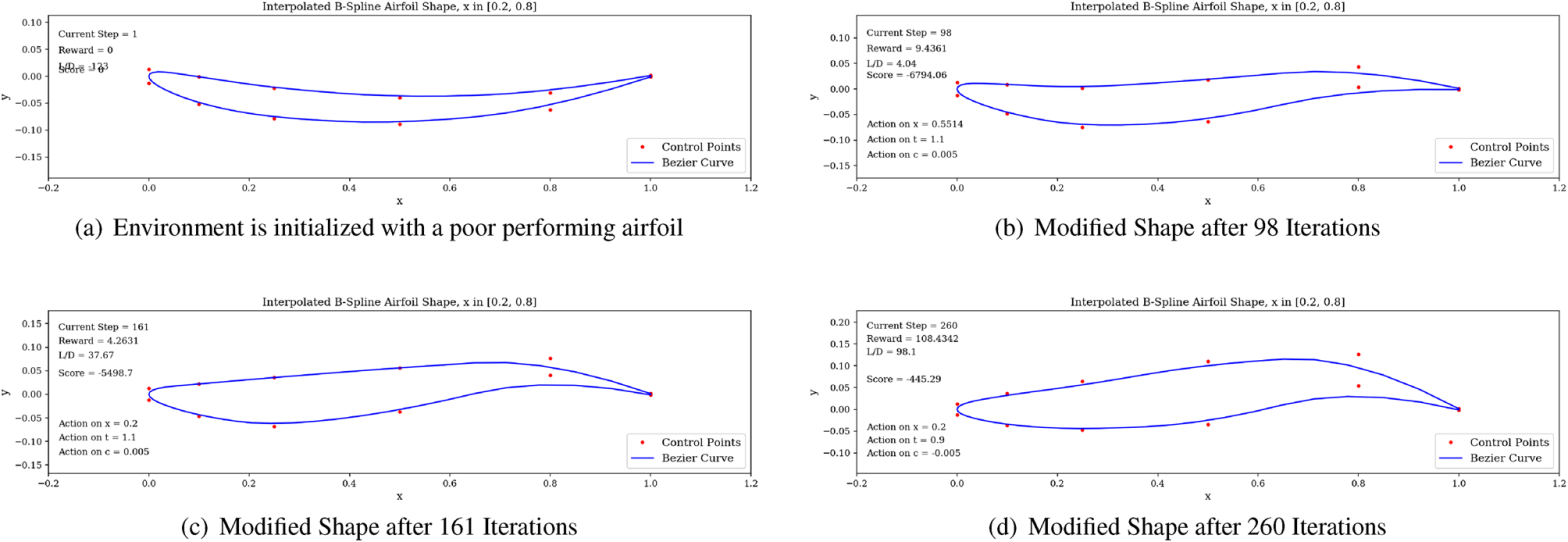
Trained agent modifies shape to produce high *L*/*D* starting with a low-performance airfoil.

An example of a high *L*/*D* shape produced by the DRL agent when starting with the flipped e58 airfoil is displayed in Fig. 18. It is interesting to notice that in this situation, the produced airfoil shares previously observed geometric characteristics, such as high camber and a drooped trailing edge, leading to a high *L*/*D* value. The trained agent is then run over one episode length in Fig. 19. By successively modifying the airfoil shape, we can observe that the agent is able to recover positive *L*/*D* values having started with a low performance airfoil. This demonstrate the correctness of the behavior learned by the agent.

**Figure 20:**
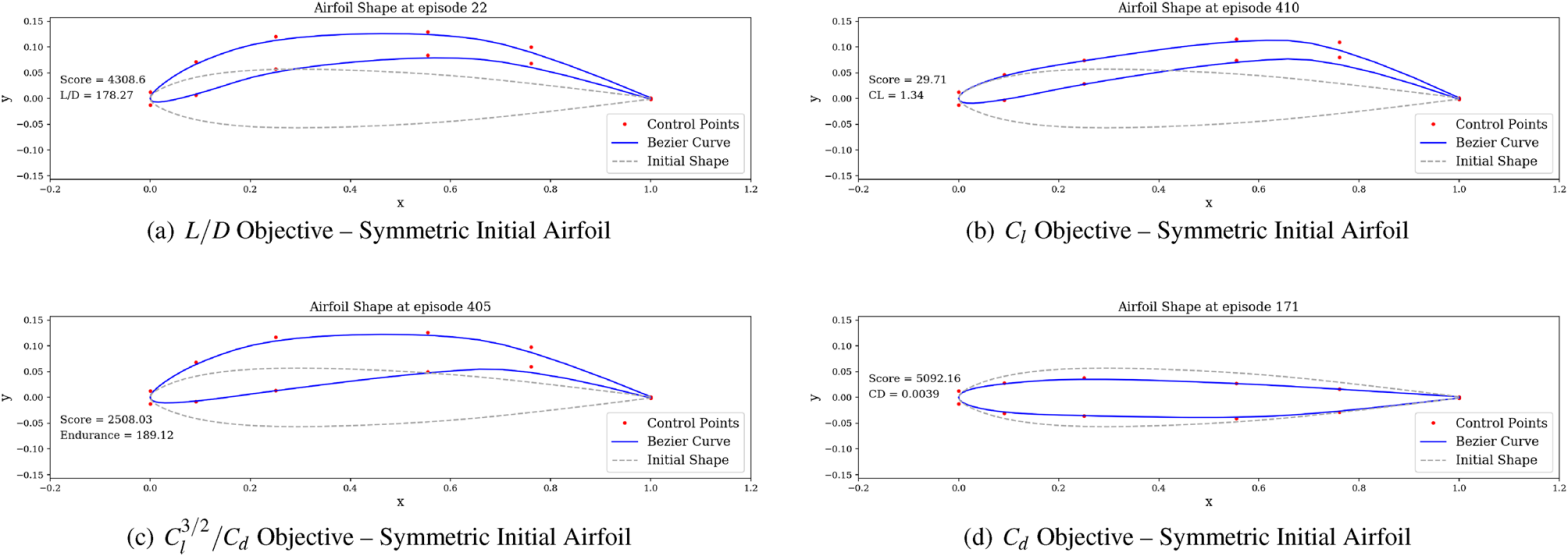
Best performing agent-produced shapes under different objectives and a symmetric initial airfoil.

**Figure 21:**
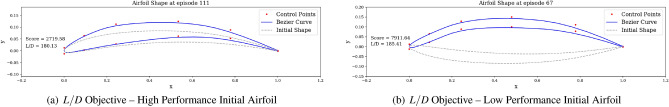
Best performing agent-produced shapes under different objectives and an asymmetric initial airfoil.

Finally, the best produced shapes (i.e., those maximizing the metric of interest) for the different objectives and tasks can now be compared, as illustrated in Figs. 20 and 21.

The results presented above demonstrate that the number of function evaluations (i.e., the number of times *Xfoil* is run and converges on a new shape proposed by the agent) depends on the task at hand. For instance, around 2,000 function evaluations were needed in Task 2, while 4,000 are needed in Task 1 and around 20,000 were required in Task 3. These differences can be explained by the ‘distance’ that exists between the starting shape and the optimal shape. In other terms, when starting with the low performing airfoil, the agent has to perform a greater number of successful steps to converge on an optimal shape, whereas when starting with an already high-performance airfoil, the agent is close to an optimal shape and requires fewer *Xfoil* evaluations to converge on an optimal shape. The number of episodes needed to reach an optimal policy, however, appears to be between 100 and 200 episodes across all tasks. Overall, when averaging across all tasks performed in this research, approximately 10,000 function evaluations were needed for the agent to converge on the optimal policy.

Having trained the RL agent on a given aerodynamic task, the designer can then draw physical insight by observing the actions the agent follows to optimize the airfoil shape. From the results presented in this research, it can be observed that high camber around the leading edge and low thickness around the trailing edge are preferred shapes to maximize *L*/*D*, given the flow conditions used here. Observing the various policies corresponding to different aerodynamic tasks, the designer can then make tradeoffs between the different aerodynamic metrics to optimize. Multi-point optimization can be achieved by including in the reward multiple aerodynamic objectives. For example, if the designer seeks to optimize both *L*/*D* and *C*_*l*_, a new definition of the reward could be: $r = (L/D_{current} + Cl_{current})-(L/D_{previous} + Cl_{previous})$ (after having normalized *L*/*D* and *C*_*l*_). However, multi-point optimization will decrease interpretability of the agent’s actions. By introducing multiple objectives in the agent’s reward, it will become more difficult for the designer to draw insight from shape changes and link those changes to maximizing a specific aerodynamic objective.

The proposed methodology enables to reduce computational costs by leveraging a data-driven approach. Having learned an optimal policy for a given aerodynamic objective, the agent can be used to optimize new shapes, without having to restart the whole optimization process. More specifically, this approach can be used to alleviate the computational burden of problems requiring high-fidelity solvers (when RANS or compressibility are required). For these problems, the DRL agent can quickly find a first optimal solution, using a low-fidelity solver. The solution can then be refined using a higher-fidelity solver and a traditional optimizer. In other words, DRL is used in this context to extract prior experience to speed up the high-fidelity optimization. As such, our approach can speed up the airfoil optimization process by very rapidly offering an initial optimal solution. Similarly to^8^, our approach can also be used directly for high-fidelity models. To accelerate convergence speeds, the DRL agent is first trained using a low-fidelity solver in order to rapidly learn an optimal policy. The agent is then deployed using a high-fidelity solver. In doing so this approach (i) reduces computational cost by shifting from a low to a high-fidelity solver to speed up the learning process, (ii) is data-efficient as the policy learned by the agent can then be followed for any comparable problem and, (iii) bears some generative capabilities as it does not require any user-provided data.

#### Comparing agent-generated shapes with existing airfoils

**Figure 22:**
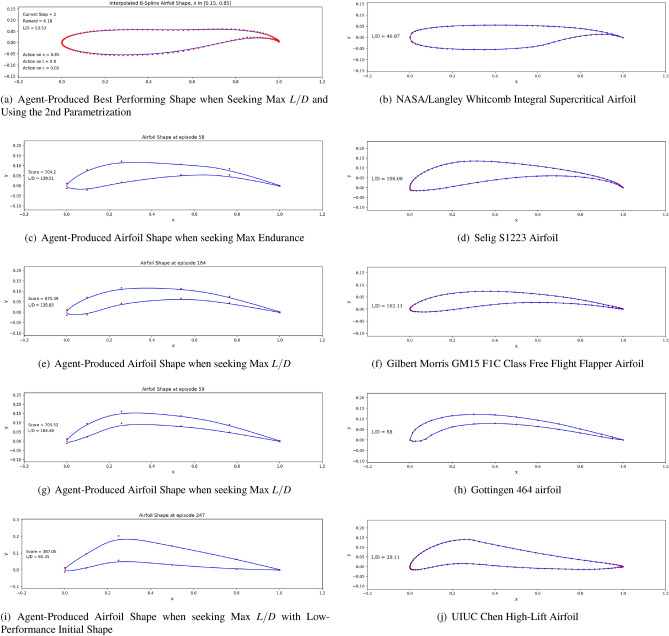
Airfoil shape comparison between agent-produced shapes and existing airfoils.

As reinforcement learning does not rely on any provided database, no preconception of what a *good* airfoil shape should look like is available to the agent. This results in added design freedom leading the agent to occasionally generate airfoil shapes that can be viewed as unusual to the aerodynamicist’s eye. In Fig. 22, we compare agent-produced shapes to existing airfoils in literature. The focus is not on the agent’s ability to produce a specific shape for given flow conditions and aerodynamic targets, but rather to illustrate the geometric similarities found on both existing airfoils and artificially-generated shapes. A strong resemblance between the agent-generated and existing airfoils can be observed. This highlights the rationality of the policy learned by the agent: having no preexisting knowledge on fluid mechanics or airfoils, an intelligent agent trained in the presented custom RL environment can generate realistic airfoil shapes.

We compare five existing airfoils to our agent-produced shapes in Fig. 22. In Fig. 22a and b, we compare the agent-produced shape to Whitcomb’s supercritical airfoil. The shared flat upper surface, cambered rear and blunt trailing edge can be noticed^51^. We then compare agent-generated shapes to existing high-lift airfoils. Here also, the geometric resemblance is noticeable, notably the shared high camber.

#### Sensitivity analysis

**Figure 23:**
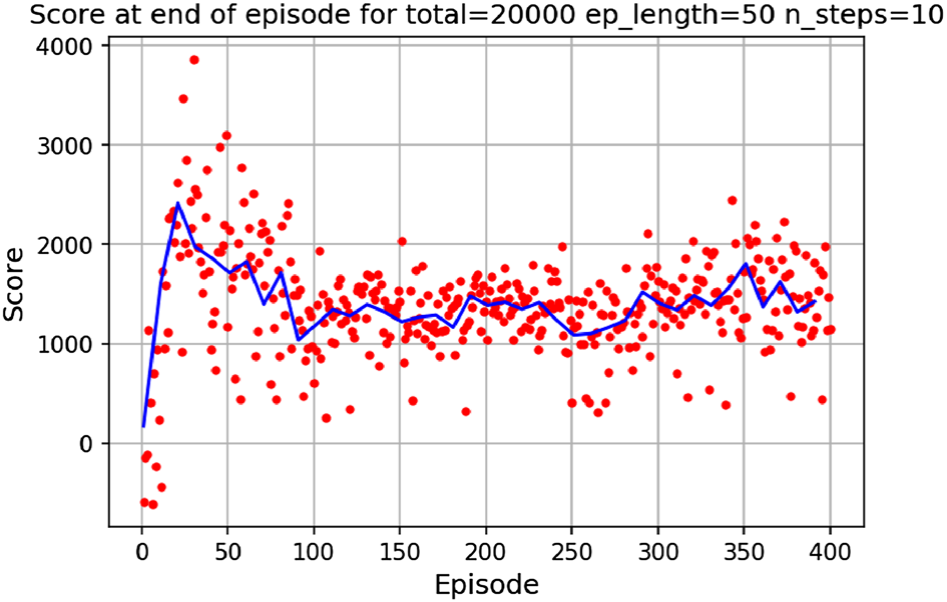
Detrimental effects of large episode lengths.

One observation was made when noticing drastic decreases in the average score at the end of episode after a first period of increase. We believe this can be explained by the fact that when the episode length is large, once the agent has learned a policy allowing to quickly (under relatively few iterations) attain high *L*/*D* values, the average score will then decrease because the agent reaches the optimal shape before the end of the episode. Within the remaining iterations before the episode ends, the agent continues to modify the shape hoping for higher performance, but reaches a limit where the shape is too extreme for the aerodynamic solver to converge, resulting in a poor reward. This would explain why we can observe on Fig. 23 a rapid increase in the score between 0 and 25 episodes, during which the agent explores various shapes and estimates an optimal policy, and a strong decrease in the score following this peak during which the agent follows the determined optimal policy and reaches optimal shapes before the episode ends.

### Benchmarking the DRL approach

The results presented above demonstrate the ability of a DRL agent to learn how to optimize airfoil shapes, provided a custom RL environment to interact with. We now compare this approach to a classical simplex method, under the same possible action conditions: starting from a symmetric airfoil, the optimizer must successively modify the shape by changing thickness and camber at selected *x* positions to achieve the highest performing airfoil in terms of *L*/*D*.

**Figure 24:**
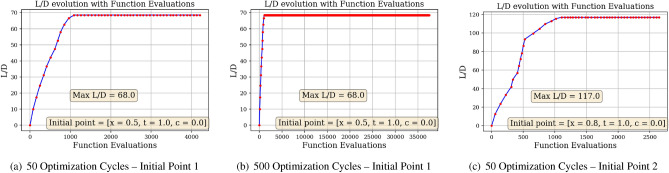
Simplex method approach–*L*/*D* increase with function evaluations for different starting points.

Here, the optimizer is based on the Nelder-Mead simplex algorithm, capable of finding the minimum of a multivariate function without having to calculate the first or second derivatives^52^. In this case, the function maps a 3-set of actions, being [select x position, change thickness, change camber] to a -*L*/*D* value. More specifically, taking the 3-set of actions as inputs, the function modifies the airfoil accordingly, evaluates the modified airfoil in *Xfoil* and outputs the associated -*L*/*D*. As the optimizer tries to minimize the- -*L*/*D* value, it searches for the 3-set that will maximize *L*/*D*. Once the optimizer finds the optimal 3-set of actions, the airfoil shape is modified accordingly and the optimizer is rerun on this new modified shape. This defines what we call *one optimization cycle*. Hence, the optimizer is tasked with the exact same optimization problem as the DRL agent: optimizing the airfoil shape to reach the highest *L*/*D* value possible by successively modifying the shape. During each optimization cycle, the optimizer evaluates the function a certain number of times. In Fig. 24, we monitor the increase in *L*/*D* with the number of function evaluations.

**Figure 25:**
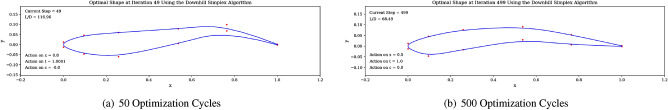
Gradient-free approach generated airfoil shapes.

In the three situations displayed, it can be observed that the value of *L*/*D* increases with the number of function evaluations. However, the converged *L*/*D* value is significantly lower than values obtained through the DRL approach. For instance, even after 500 optimization cycles (i.e., 500 shape modifications and over 30,000 function evaluations), the optimizer is unable to generate an airfoil having *L*/*D* over 70. We know that this value of *L*/*D* is not a global optimum, as an *L*/*D* of at least 160 can be reached with the Eppler 58 airfoil from the *UIUC database*^41^. Thus, it seems that the simplex algorithm has converged on a local minimum. Furthermore, as demonstrated in Fig. 24a and c, the converged *L*/*D* value found by the optimizer is highly dependent on the initial point. The airfoil shapes generated using the simplex method can be found in Fig. 25.

In Table 3, we compare the converged *L*/*D* values, number of iterations and run times of the simplex method and DRL approach. In both approaches, the agent or optimizer can modify the airfoil 60 times. Although the number of iterations and run time are lower for the simplex method, the converged *L*/*D* value is far lower compared to the DRL approach.

**Table 3:** Simplex method versus DRL approach comparison.

	Simplex	DRL
Converged *L*/*D*	68	171
Function Evaluations / Learning Iterations	5000	12,000
Run time (in seconds)	124	326

This rapid simplex approach to the airfoil shape optimization problem highlights the benefits and capabilities of the presented DRL approach. First, the DRL approach seems less prone to convergence on local minima, as very high values of *L*/*D* can be achieved. Second, once the DRL agent has learned the optimal policy during a training period, it can be applied directly to any new situation whereas the simplex approach will require a whole optimization process for each new scenario encountered.

## Conclusion

This research presents a Deep Reinforcement Learning (DRL) approach to the airfoil shape optimization problem. This approach is motivated by (i) the recent success of data-driven approaches at solving complex aerodynamic problems, where non-linearity and high-dimensionality are inherent, and (ii) the exploratory capabilities of reinforcement learning. Airfoil design is formulated as a Markov decision process, making RL a suitable candidate for this problem. A custom RL environment is developed integrating a low-fidelity aerodynamic solver. By interacting with the provided environment, the DRL agent learns how to best modify an airfoil shape to increase its aerodynamic performance. The research investigates the impact of different performance objectives on the learning process such as maximizing *L*/*D*, maximizing *C*_*l*_, maximizing endurance and minimizing *C*_*d*_. The impact of the initial airfoil observed by the agent is also investigated. Results demonstrate the learning capabilities of DRL in all cases. Specifically, the agent is able to learn an optimal policy within a limited number of learning iterations and is capable of producing airfoil shapes exhibiting remarkably high aerodynamic performance. When compared to a classical simplex approach, the DRL method proves more efficient at exploring the design space and is able to converge on higher performing airfoils.

The contributions of this research to machine learning-based approaches for airfoil shape optimization problem are twofold. First, we demonstrate that RL can successfully be applied to a physics-based problem, where RL is traditionally reserved to game-based tasks. We show that the generative and exploratory capabilities of RL can be leveraged to generate optimal airfoils, as opposed to supervised learning approaches which rely on, and are limited to provided data. Secondly, our approach provides the agent with enhanced action freedom, being able to modify the thickness and camber of the airfoil at local chordwise positions, where previous investigations were limited by the agent’s action space. Finally, the impact of other crucial parameters, such as the aerodynamic objective and the starting airfoil, are investigated.

Further efforts are needed to complement this DRL approach to airfoil shape optimization. An increased number of control points accessible to the agent would allow for greater flexibility and potentially higher performing agent-generated airfoils. Coupling with a more robust aerodynamic solver is needed to limit the amount of unconverged shapes as well as provide higher fidelity regarding the calculated performance metrics. A more complex approach to geometric abnormality constraints could also be implemented into the proposed RL-based optimization to improve convergence and increase the efficiency of the aerodynamic shape optimization task. Investigating the impact of an increased design space by adding the airfoil’s angle-of-attack to the agent’s action-space would also be of interest. Finally, training the DRL agent over a vast number of initial airfoils would increase the knowledge acquired by the agent and would further enhance the capabilities of the DRL approach.

## Data Availability

The code alongside the datasets used and generated during the current study are available from the corresponding author on reasonable request.
